# Intrinsic T cell glutaminolysis promotes autoimmunity in lupus-prone mice

**DOI:** 10.1172/jci.insight.192286

**Published:** 2025-09-16

**Authors:** Seung-Chul Choi, Yong Ge, Milind V. Joshi, Damian Jimenez, Abigail Castellanos Garcia, Cassandra LaPlante, Lauren T. Padilla, Chaoyu Ma, Nu Zhang, Jeffrey C. Rathmell, Mansour Mohamadzadeh, Laurence Morel

**Affiliations:** 1 Department of Microbiology, Immunology, and Molecular Genetics, University of Texas Health San Antonio (UTHSA), San Antonio, Texas, USA.; 2 South Texas Veterans Health Care System, San Antonio, Texas, USA.; 3 Ben May Department for Cancer Research and Ludwig Center at the University of Chicago, Chicago, Illinois, USA.

**Keywords:** Autoimmunity, Immunology, Adaptive immunity, Amino acid metabolism, T cells

## Abstract

Glutaminolysis is enhanced in T cells of patients with lupus and in Tfh cells, a critical subset of CD4^+^ T cells that provide help to autoreactive B cells, in lupus mice. Glutaminolysis inhibitors reduced lupus activity in association with a decreased frequency of Th17 cells in mice. Here, we thought to determine the role of glutaminolysis in murine Tfh cells. The pharmacological inhibition of glutaminolysis with DON reduced the expression of the critical costimulatory molecule ICOS on lupus Tfh cells, in association with a reduction of autoantibody production and B cell differentiation markers. Accordingly, profound transcriptomic and metabolic changes, including a reduction of glycolysis, were induced by DON in lupus Tfh cells, whereas healthy Tfh cells showed minor changes. The T cell–specific genetic inhibition of glutaminolysis largely phenocopied the effects of DON on Tfh cells and B cells in an autoimmune genetic background with minor changes in Tfh and B cells in healthy controls. Furthermore, we showed that T cell–specific glutaminolysis inhibition impaired T-dependent humoral responses in autoimmune mice as well as their Tfh response to a viral infection. Overall, these results suggest that lupus Tfh cells have a greater intrinsic requirement of glutaminolysis for their helper functions.

## Abstract 2

This study showed that the pharmacological and T cell-specific inhibition of glutaminolysis impaired the function of follicular helper T cells in lupus.

## Introduction

Glutamine is required for T cell proliferation, serving as a precursor for nucleotide synthesis. In addition, glutaminolysis in the mitochondria converts glutamine into glutamate by glutaminases, including glutaminase 1 encoded by *Gls1*, then into α-ketoglutarate (αKG), which enters the TCA cycle and contributes to oxidative phosphorylation (OXPHOS) and ATP production. Glutamate is also a precursor for the biosynthesis of glutathione, an essential component of redox homeostasis that is critically reduced in lupus T cells (1). αKG is a critical regulator of chromatin accessibility as a cofactor of histone and DNA demethylases (2).

*Gls1* deletion in T cells impaired Th17 cell differentiation and altered Th1 cell functions, in association with epigenetic changes (3). ICER, a transcription factor that promotes Th17 differentiation, directly transactivates *Gls1* expression (4) and, thus, induces a metabolic program that supports Th17 cells. Peripheral blood mononuclear cells (PBMCs) from patients with systemic lupus erythematosus (SLE) and splenocytes from lupus-prone MRL/lpr mice present high levels of glutamate and *GLS1* expression (5). Treatment of MRL/lpr mice with the *Gls1* inhibitors CB839 (5) or BPTES (6) ameliorated disease by reducing the frequency of Th17 cells, a major pathogenic subset in this model. Paradoxically, *Gls1* inhibition reduced glycolysis, which is required for Th17 differentiation (7), by decreasing the expression of HIF1α (6). Other mechanisms by which glutaminolysis contributes to Th17 differentiation have been identified in other models (3). Elevated expression of *Gls1* in psoriasis promotes the expression of IL-17A by enhancing histone acetylation at the *Il17a* promoter (8). In addition, the accumulation of TCA cycle–generated glutaminolysis intermediates that influence chromatin accessibility is tightly controlled in Th17 cells by LBK1 that senses mitochondrial integrity (9). Glutamine-driven OXPHOS also increases apoptosis resistance and cytotoxicity of Th17 cells against tumors, which could otherwise not be achieved by glycolysis (10). Pharmacological inhibition of glutaminolysis with 6-diazo-5-oxo-L-norleucine (DON) prevented allograft rejection by reducing T cell activation (11). On the other hand, DON treatment upregulated OXPHOS in tumor infiltrating CD4^+^ T cells and promoted an effector long-lived phenotype (12).

Glutaminolysis also contributes critically to B cell functions. TLR9 signaling and type I IFN induce the differentiation of human B cells into plasmablasts by orchestrating glutamine-dependent OXPHOS in vitro (13). Similar to CD4^+^ T cells (14), B cells from patients with SLE are characterized by an enhanced OXPHOS (13). Since activation through TLR9 ligands and type I IFN is a characteristic feature of lupus B cells, these results suggest that targeting glutaminolysis may reduce plasmablast differentiation and autoantibody production in SLE. However, plasma cells were not affected in MRL/lpr mice treated with CB839 (5). Glutaminolysis is also required by human B10 cells to exert immunosuppression (15). However, CB839 increased the frequency of B10 cells in MRL/lpr mice (5). Thus, the role of glutaminolysis in B cells is complex and may be context dependent.

We recently investigated the role of glutaminolysis in the expansion of CD4^+^ Tfh cells and germinal center (GC) B cells leading to the production of autoantibodies in the lupus-prone triple congenic (TC) B6.*Sle1*.*Sle2*.*Sle3* mouse model. Treatment of TC mice with DON strongly reduced anti-dsDNA IgG levels, virtually eliminated GCs, and reduced the frequency of GC B cells (16). In vitro, however, DON did not alter the differentiation of purified TC or C57BL/6 (B6) control B cells into antibody-secreting cells (ASCs) under either T-dependent and T-independent conditions (17), suggesting that DON may exert an extrinsic effect on B cells in vivo. DON treatment did not reduce the frequency of Tfh cells in TC mice, but it reduced their ICOS and BCL-6 expression (16). In addition, DON did not reduce in vitro Tfh polarization in either TC or B6 purified CD4^+^ T cells (18). A slight reduction in the frequency of Tfh cells and GC B cells was observed in MRL/lpr mice treated with CB839 (5). These results suggest that glutaminolysis is not required for Tfh cell differentiation, but it may affect their function, as it does for Th1 cells (3). In support of this hypothesis, we showed that TC Tfh cells exhibit a differential expression of glutamine transporters as well as an enhanced glutamate metabolism compared with B6 controls (19).

To gain mechanistic insights on the role of glutaminolysis in lupus Tfh cells, we first analyzed the effect of its pharmacologic inhibition with DON in the (NZW x BXSB.Yaa)F1 model of the lupus, hereafter referred to as W.Yaa, compared with healthy control B6 mice. In the W.Yaa model, the *Yaa* duplication of the *Tlr7* locus on the Y chromosome controls pathogenesis largely through TLR7/type 1 IFN signaling (20), a pathway critically involved in SLE (21). This includes the extrafollicular differentiation of autoantibody-producing cells, with a robust expansion of age-related B cells (ABCs) and extrafollicular Th (Texfh) cells (22), 2 subsets corresponding to the DN2 B cells (23) and Tph cells (24) expanded in patients with SLE. The W.Yaa model is, thus, highly relevant to elucidate the mechanisms rewiring cellular metabolism to potentially control autoreactive Tfh cells and mitigate disease progression. We have previously demonstrated that the pharmacological inhibition of glycolysis with 2-deoxyglucose (2DG) completely reversed the autoimmune manifestations and renal pathology in W.Yaa mice (25). Here, we show that DON reduced autoantibody production and the frequency of ABCs and plasma cells in W.Yaa mice. DON had no effect on the frequency of W.Yaa Tfh and Texfh cells, but it reduced the expression of ICOS and OX-40, which are critical regulators of Tfh cell differentiation, maintenance, and function (26, 27). Comparatively, DON had little effect on B6 mice. DON also uniquely modulated the transcriptome and metabolome of W.Yaa Tfh cells. To investigate the T cell–intrinsic role of glutaminolysis on Tfh cells and to eliminate potential off target effects of DON, we bred *Gls1* deficiency on T cells in B6.*Sle1b* mice, a congenic strain derived from the NZM2410 lupus model carrying mutations in 6 SLAM family genes (28), which functionally regulate Tfh cells and GC B cells (29, 30). B6.*Sle1b* mice produce anti-dsDNA IgG autoantibodies (31) and robustly expend GC B cells (32) and Tfh cells (33). Here, we show that the genetic inhibition of glutaminolysis in T cells reduced both spontaneous and induced autoantibody production, and phenocopied most of the cellular phenotypes induced by the DON treatment in W.Yaa mice. Furthermore, *Gls1* deficiency in T cells uniquely impaired the response to protein immunization as well as to a viral infection in B6.*Sle1b* as compared with B6 mice. Thus, these results suggest that lupus CD4^+^ T cells have a higher intrinsic demand for glutaminolysis, which supports Tfh functions and indirectly promotes autoantibody production.

## Results

### DON reduced autoantibody production and altered selective GC B cell and CD4^+^ T cell phenotypes in W.Yaa mice.

**Figure 1:**
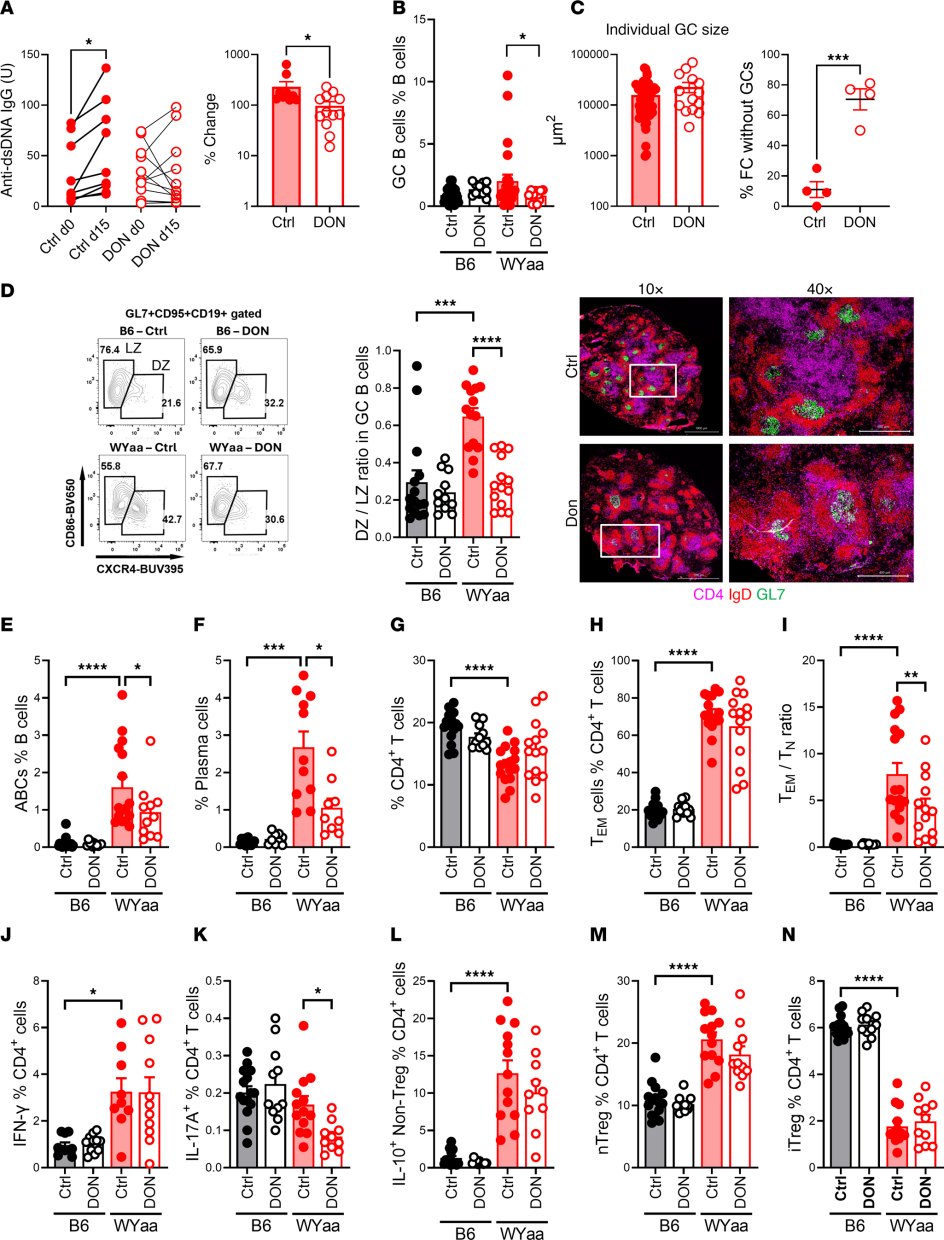
DON reduced autoantibody production and altered selective GC B cell and CD4^+^ T cell phenotypes in W.Yaa mice. (**A**) Serum anti-dsDNA IgG measured before and after treatment. Left: Paired unit values for each mouse compared with a paired *t* test. Right: Percent change after 2 weeks, mean ± SEM, Mann-Whitney *U* test. *n* = 9 controls and 12 DON-treated. (**B**) Frequency of GC B cells compared with a Fisher’s exact test. (**C**) Spleen GC histology: Individual GC size (left) and frequency of follicles without GC (right) in untreated control and DON-treated W.Yaa mice (*n* = 4) compared with a *t* test. Representative images of GCs in untreated (top) and DON-treated (bottom) spleens shown as GL7^+^IgD^–^ areas. The 40× images correspond to the boxed areas on the 10× images. (**D**) GC B cell DZ/LZ ratio with representative FACS plots. (**E**–**H**) Frequency of ABCs (**E**), plasma cells (**F**), total CD4^+^ T cells (**G**), and Tem cells (**H**). (**I**) Tem/Tn ratio. (**K**–**L**) Frequency of IFN-γ^+^ (**J**), IL-17A^+^ (**K**), and IL-10^+^ (**L**) in FOXP3-negative CD4^+^ T cells. (**M** and **N**) Frequency of nTreg (**M**) and iTreg (**N**) cells. Mean ± SEM, *n* = 11–25 compared with Dunnet’s, Šídák’s (**E**) or Kruskal-Wallis (**K**) multiple-comparison tests, and with a Mann-Whitney test (**B**). *P < 0.05; ***P < 0.001; ****P < 0.0001.

Treatment of W.Yaa mice with DON for 2 weeks reduced the production of anti-dsDNA IgG, which increased in untreated mice (Figure 1A). A similar trend was observed after a 4-week treatment (Supplemental Figure 1A; supplemental material available online with this article; https://doi.org/10.1172/jci.insight.192286DS1), which significantly reduced renal injury evaluated by pathology score, glomerular size, or complement C3 deposits (Supplemental Figure 1, B–E). A high frequency of CD19^+^CD4^–^GL7^+^CD95^+^ GC B cells (defined as greater than the mean + 1 SD from B6 control mice) was observed in 9 of 25 W.Yaa mice but in none of the W.Yaa mice treated with DON (Fisher’s exact test, *P* = 0.0341; Figure 1B and Supplemental Figure 2). In addition, W.Yaa mice develop visible GCs in almost every spleen follicle, and these GCs were largely eliminated by the DON treatment. However, the size of the remaining GCs was similar to that of control mice (Figure 1C). Moreover, the higher dark zone to light zone (CXCR4^hi^CD86^lo^ DZ/CXCR4^lo^CD86^hi^ LZ) ratio of the W.Yaa GC B cells was reduced to B6 levels by DON (Figure 1D), suggesting that DON altered the GC B cell dynamics. As expected with the *Tlr7* duplication, W.Yaa mice presented a large population of CD19^+^CD4^–^CD93^–^CD5^lo/–^CD21^–^CD23^–^CD11c^+^T-bet^+^ ABCs, whose frequency was reduced by DON (Figure 1E). Finally, DON reduced the frequency of splenic CD138^+^IgD^–^ plasma cells (Figure 1F). This could result from the reduced ABC population, the altered GC B cell development, and a direct effect of DON on plasma cells.

W.Yaa mice presented a decreased frequency of CD4^+^ T cells that did not change with DON (Figure 1G). There was a trend for DON reducing the high frequency of W.Yaa effector memory T cells (CD4^+^CD44^+^CD62L^–^ Tem), leading to a significantly decreased Tem/CD4^+^CD44^–^CD62L^+^ naive T (Tn) cell ratio, which is typically elevated in lupus-prone mice (Figure 1, H and I). DON had no effect on the high frequency of IFN-γ–producing CD4^+^ T cells, but it reduced the small population of IL-17A–producing T cells (Figure 1, J and K), which is consistent with the effect of glutaminolysis inhibition reported by others on Th1 and Th17 cells (3, 6). IL-10–producing FOXP3^–^ (non-Treg) CD4^+^ T cells are expanded in lupus, including in W.Yaa mice, and the frequency of these cells was not affected by DON (Figure 1L). Finally, W.Yaa mice presented skewed frequencies of thymic-derived “natural” CD4^+^FOXP3^+^Helios^+^ nTregs and peripherally induced CD4^+^FOXP3^+^Helios^–^ iTregs, which were also not affected by DON (Figure 1, M and N).

**Figure 2:**
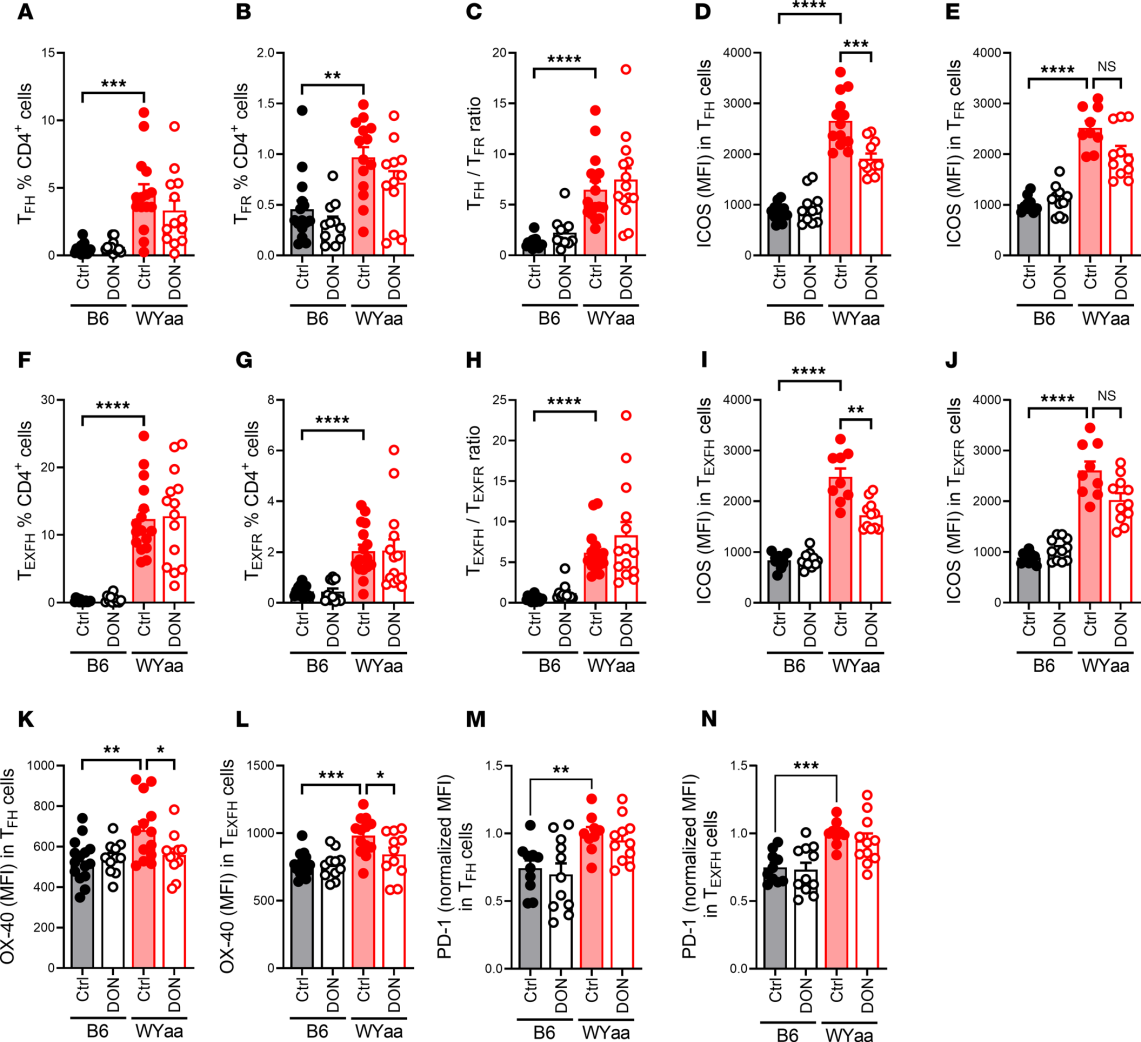
DON reduced ICOS and OX-40 expression on W.Yaa Tfh and Texfh cells with minimal effect on B6 cells. (**A** and **B**) Frequency of Tfh and Tfr cells. (**C**) Tfh/Tfh ratio. (**D** and **E**) ICOS expression of Tfh and Tfr cells. (**F** and **G**) Frequency of Texfh and Texfr cells. (**H**–**J**) Texfh/Texfr ratio. ICOS expression of Texfh and Texfr cells. (**K** and **L**) OX-40 expression on Tfh and Texfh cells. (**M** and **N**) PD-1 expression on Tfh and Texfh cells. Values were normalized to the W.Yaa mean value for each cohort. Mean ± SEM, *n* = 10–15 compared with Šídák’s, Dunnett’s (**K** and **L**) or Kruskal-Wallis (**B** and **H**) multiple-comparison tests. *P < 0.05; **P < 0.01; ***P < 0.001; ****P < 0.0001.

As the TC lupus-prone model (16), W.Yaa mice presented an expanded population of CD4^+^CD44^+^FOXP3^–^CXCR5+BCL6^+^CD162^lo/–^PD-1^+^ Tfh cells as well as follicular Tregs (CD4^+^CD44^+^FOXP3^+^CXCR5^–^BCL6^–^CD162^lo/–^PD-1^+^ Tfr), with a skewed Tfh/Tfr ratio, none of which were altered by DON (Figure 2, A–C). However, as in TC mice, DON reduced the high expression of ICOS on Tfh and Tfr cells (Figure 2, D and E). Consistent with the high frequency of ABCs, W.Yaa mice developed an expanded population of CD4^+^ Texfh (CD4^+^CD44^+^FOXP3^–^CXCR5^–^BCL6^–^CD162^lo/–^PD-1^+^ Texfh) cells and their corresponding FOXP3^+^ regulatory subset of CD4^+^CD44^+^FOXP3^+^CXCR5^–^BCL6^–^CD162^lo/–^PD-1^+^ Texfr cells, with their frequency or ratio also not affected by DON (Figure 2, F–H). DON also reduced ICOS expression on Texfh and Texfr cells (Figure 2, I and J), as well as on Tn cells, Tem cells, and Tregs (Supplemental Figure 3, A-D), suggesting a requirement for glutaminolysis for ICOS expression.

TCR activation triggers *Icos* transcription (34). Consistent with a stronger TCR signaling in lupus Tfh cells, *Icos* message expression was higher in W.Yaa than in B6 Tfh cells, and it was decreased by DON (Supplemental Figure 3E) in a similar pattern as ICOS protein expression (Figure 2D). ICOS is degraded through ubiquitination by CBL/CBL-B, which are expressed at lower levels by CD4^+^ T cells from patients with SLE (35). *Cbl* expression was lower in W.Yaa than B6 Tfh cells and there was a similar trend for CBL protein in W.Yaa CD44^+^ CD4^+^ T cells, but neither message nor protein were affected by DON (Supplemental Figure 3, F, G, and J). *CBL-B* expression was similar between W.Yaa than in B6 Tfh cells, while CBL-B protein expression was lower in W.Yaa CD44^+^ CD4^+^ T cells relative to B6, but it was not affected by DON (Supplemental Figure 3, H–J). These results suggest that the increased ICOS expression in W.Yaa as compared with B6 Tfh cells is largely controlled at the transcriptional level, with potential additional involvement of a decreased CBL/CBL-B expression. The inhibition of glutaminolysis reduced ICOS expression at the transcriptional level, potentially through epigenetic modification—and potentially at the posttranscriptional level—but not through increased protein degradation by CBL/CBL-B. DON also reduced OX-40 expression on Tfh, Texfh, and Tfr cells (Figure 2, K and L, and Supplemental Figure 3, K and L). The downregulation of OX-40 by DON was, however, more selective than ICOS, with no change observed in Texfr cells, Tn cells, Tem cells, Tregs (Supplemental Figure 3, M–P). Finally, PD-1 expression sustains Tfh cell numbers and promotes GC B cell differentiation into long-lived plasma cells (36). Accordingly, PD-1 expression was higher on W.Yaa than on B6 Tfh and Texfh cells (Figure 2, M and N). However, PD-1 expression was not affected by DON. Overall, DON selectively altered W.Yaa CD4^+^ T cells, including the expression of critical costimulatory receptors, namely ICOS and OX-40, on Tfh-related subsets, suggesting that glutaminolysis altered their maintenance and/or function, while it showed minimal effects on healthy B6 CD4^+^ T cells.

### Transcriptomic reprogramming of W.Yaa Tfh cells by DON.

**Figure 3:**
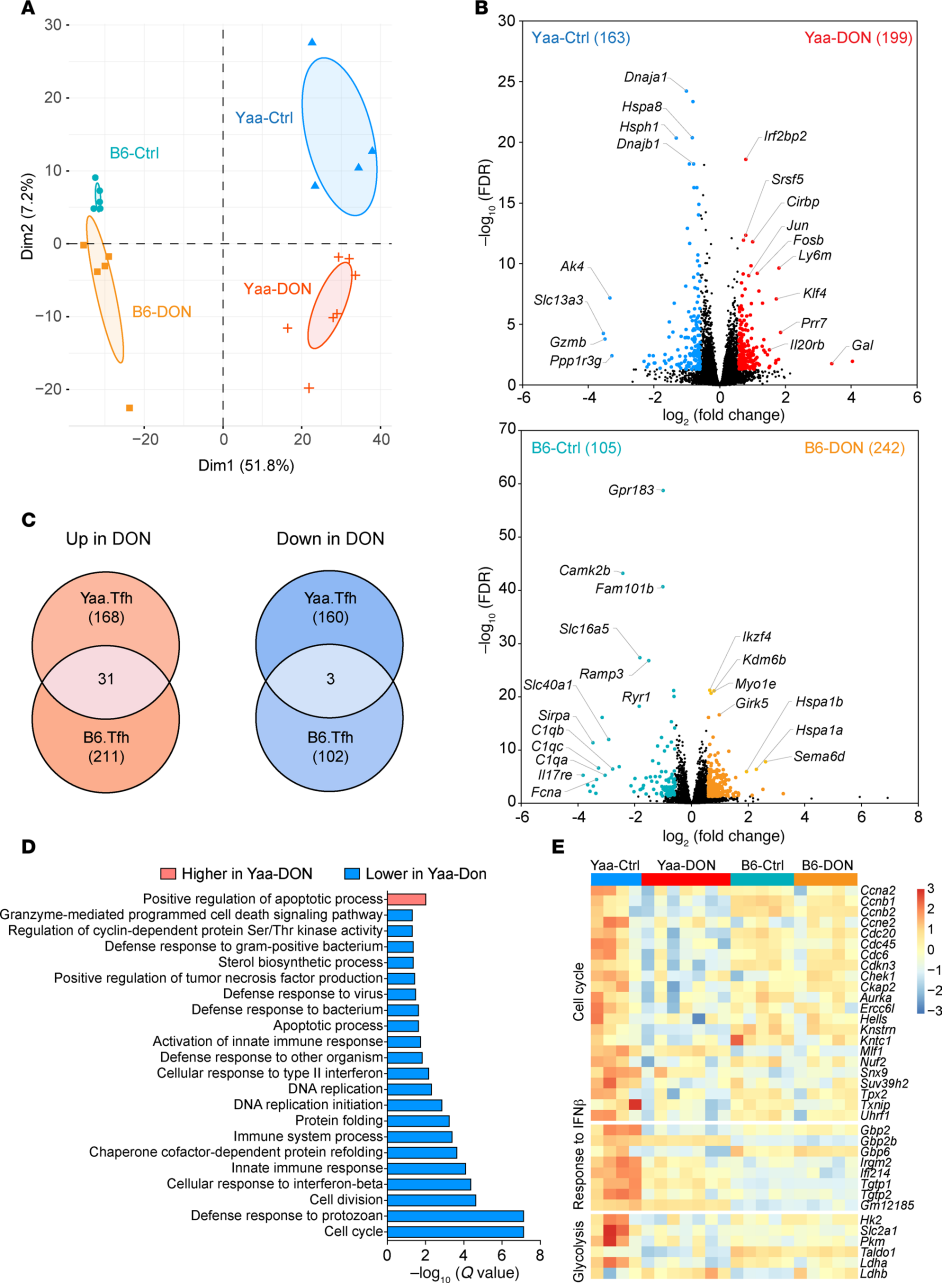
DON differentially regulates the transcriptomic programming of W.Yaa Tfh cells. (**A**) PCA plot of the transcriptomes of Tfh cells from W.Yaa and B6 mice treated with DON or untreated controls (*n* = 4–7 per group). (**B**) RNA-Seq results depicted as volcano plots showing DEGs (fold change > 1.5, FDR < 0.05) overexpressed in DON-treated (red) and control (blue) groups. The number of DEGs upregulated in indicated groups is shown in parentheses. (**C**) Venn diagrams depicting the unique effect of DON on W.Yaa Tfh cells. (**D**) DAVID Gene Ontology (GO) analysis, related to biological process, showing upregulated or downregulated pathways in W.Yaa Tfh cells by DON treatment. (**E**) Heatmap of DEGs related to cell cycle, response to IFN-β, and glycolysis. Each column represents a mouse.

To unbiasedly investigate the cellular response to DON treatment, an RNA-Seq analysis was performed on splenic Tfh cells isolated from DON-treated (Yaa-DON) and untreated W.Yaa (Yaa-Ctrl) mice compared with DON-treated (B6-DON) and untreated B6 mice (B6-Ctrl). Tfh cells were sorted as CD4^+^CD44^+^PD-1^+^PSGL-1^lo^ cells (37), which include Texfh, Tfr, and Texfr cells (Supplemental Figure 6). DON treatment resulted in a markedly transcriptomic reprogramming of splenic Tfh cells isolated from both mouse strains, as demonstrated by principal component analysis (PCA) (Figure 3A). Differentially expressed genes (DEGs) downregulated by DON in W.Yaa Tfh cells included several heat shock proteins (e.g., *Hspa8*, *Hsph1*, *Dnaja1*, *Dnajb1*), which are often triggered by cellular stress and inflammation (Figure 3B). Some of these genes, including *Hspa1a* and *Hspa1b*, were also activated in splenic Tfh cells derived from B6 mice after DON treatment. Furthermore, few DEGs were shared between W.Yaa and B6 Tfh cells (Figure 3C), highlighting a specific effect of DON on autoimmune Tfh cells. Gene set enrichment analysis (GSEA) shows that DON mainly inhibited the signaling pathways related to cell cycle and type I IFN response in W.Yaa Tfh cells (Figure 3D). Accordingly, the expression of a group of cell cyclin and cell division genes, as well as IFN-stimulated genes (ISGs), which demonstrated the highest expression in untreated W.Yaa Tfh cells, was notably reduced in W.Yaa Tfh cells by DON to the level of B6 Tfh cells (Figure 3E). In addition, genes involved in glycolysis, including hexokinase 2 (*Hk2*), glucose transporter (*Slc2a1/Glut1*), and pyruvate kinase (*Pkm*), were also inhibited by DON. Lactate dehydrogenase A (*Ldha*), critically supporting glycolysis and T cell differentiation (38), was also suppressed by DON, while its isoenzyme *Ldhb*, whose deletion in T cells enhances glycolysis, was increased (Figure 3E), indicating that DON may reprogram the glycolysis to potentially control T cell differentiation.

### DON uniquely altered the metabolism of W.Yaa Tfh cells.

**Figure 4:**
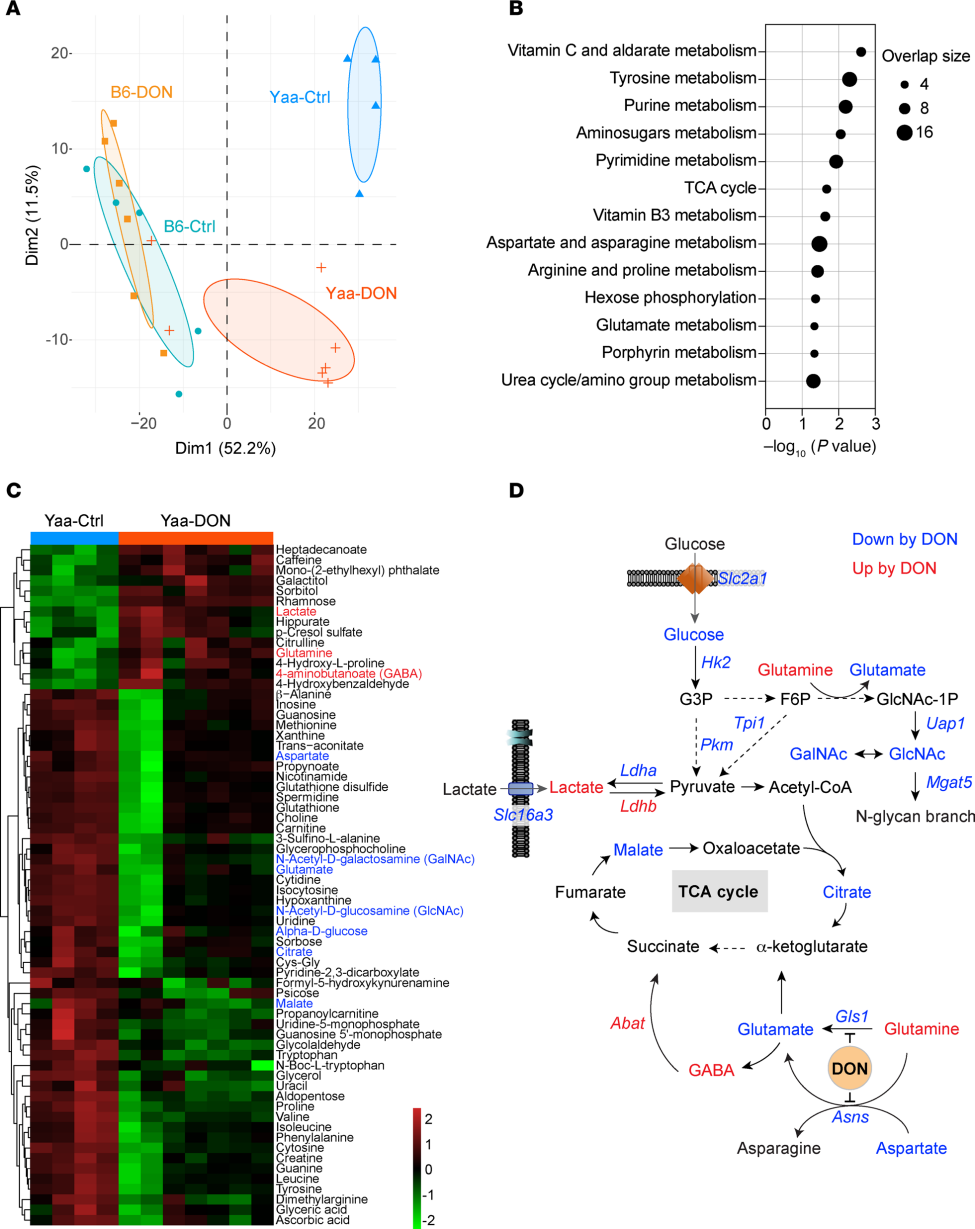
DON inhibits glycolysis and metabolically controls cell proliferation of autoimmune Tfh cells. (**A**) PCA plot of metabolite features in Tfh cells from W.Yaa and B6 mice after DON treatment versus untreated controls (*n* = 4–7/group). (**B**) Significant metabolic pathways in Tfh cells from W.Yaa mice treated with DON and control. (**C**) Heatmap of differentially enriched metabolites in W.Yaa versus W.Yaa-DON Tfh cells. (**D**) Transcriptional and metabolic integration illustrating the suppression of glycolysis and *N*-glycosylation activities in W.Yaa Tfh cells by DON. Gene (italic) and metabolites with higher and lower levels in DON group compared with controls are highlighted in red and blue, respectively, and shown on the heatmap in **C**.

Next, we analyzed the metabolome of Tfh cells isolated from the cohorts of W.Yaa and B6 mice described above. While no segregation was observed between the 2 groups of B6 Tfh cells, DON treatment significantly shifted the metabolome of W.Yaa Tfh cells toward a B6-like metabolic phenotype (Figure 4A). While no pathway significantly differed between DON-treated and untreated B6 mice, amino acid (e.g., glutamate and arginine) and nucleotide metabolism were the primary pathways affected by DON in W.Yaa Tfh cells (Figure 4B). Increased glutamine and decreased glutamate levels were evident in Tfh cells from DON-treated W.Yaa mice. (Figure 4C). γ-Aminobutyric acid (GABA), reported to inhibit T cell autoimmunity and the development of inflammatory T cells (39), was enriched in the Yaa-DON group (Figure 4C). One of the major functions of respiration in proliferating cells is to provide electron acceptors for aspartate biosynthesis (40). Moreover, glutamine is the primary source for aspartate biosynthesis in highly proliferating cells, such as cancer cells (41). Interestingly, aspartate and a number of nucleobases (cytosine, guanine, and uracil), nucleosides (guanosine, inosine, uridine and cytosine), and nucleotides (uridine 5′-monophosphate and guanidine 5′-monophosphate) were strongly reduced in Tfh cells from DON-treated W.Yaa mice (Figure 4C), potentially resulting in a global suppression of cellular metabolic activities, as demonstrated by massive reductions in amino acids, including alanine, valine, proline, leucine, isoleucine, tyrosine, phenylalanine, and tryptophan (Figure 4C).

Transcriptional and metabolic integration correlatively uncovered a controlled glycolysis program in autoimmune Tfh cells via DON treatment, which was accompanied by a slight reduction in TCA intermediates (e.g., malate, citrate) (Figure 4D). Intriguingly, both N-acetyl-D-galactosamine (GalNAc) and N-acetyl-D-glucosamine (GlcNAc), which are involved in protein N-glycosylation and regulation of lymphocyte adhesion, maturation, and activation (42), were reduced in DON-treated Tfh cells. Furthermore, b-1,6 N-acetyl-glucosaminyltransferase 5 (*Mgat5*), which initiates GlcNAc-b-1,6 branching at N-glycans, serving as ligands for galectins known to enhance T cell proliferation (43), was also reduced, suggesting the potential metabolic control of TCR signaling and cell proliferation.

### Glutaminolysis inhibition in T cells reduced spontaneous autoimmunity.

**Figure 5:**
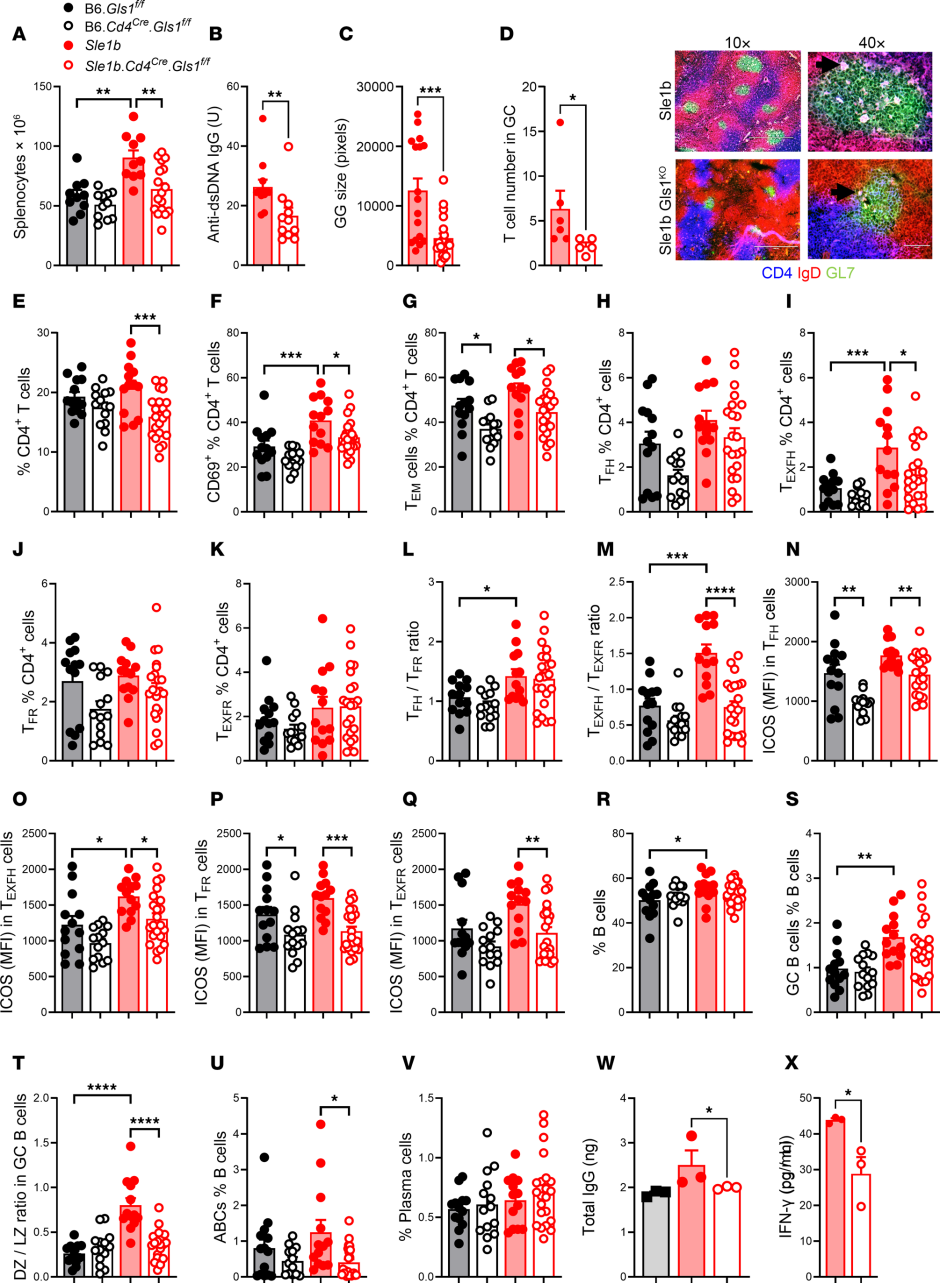
*Gls1* CD4-KO reduced the production of autoantibodies in aged *Sle1b* mice with selective effects on CD4^+^ T cells and B cells. (**A**) Splenocyte numbers. (**B**) Serum anti-dsDNA IgG. (**C** and **D**) Size of GCs in the spleen of 3 mice per group with representative images (10×) GCs in *Sle1b* (top) and *Sle1b* *Gls1* CD4-KO (bottom) spleens shown as GL7^+^IgD^–^ areas. The 40× images on the right show magenta CD4^+^ T cells inside the GCs and the corresponding quantification in the GCs in which T cells could be clearly counted. (**E**–**K**) Frequency of CD4^+^ T cells (**E**), CD69^+^CD4^+^ (**F**), Tem (**G**), Tfh (**H**), Texfh (**I**), Tfr (**J**), and Texfr (**K**) cells. (**L** and **M**) Tfh/Tfr (**L**) and Texfh/Texfr (**M**) ratios. (**N**–**Q**) ICOS expression on Tfh (**N**), Texfh (**O**), Tfr (**P**), and Texfr (**Q**) cells. (**R** and **S**) Frequency of total B cells (**R**) and GC B cells (**S**). (**T**) DZ/LZ ratio in GC B cells. (**U** and **V**) Frequency of ABCs (**U**) and plasma cells (**V**). *n* = 13–22, 9- to 12-month-old mice. (**W**–**X**) Supernatants of cocultures between *Sle1b* or *Sle1b* *Gls1* Tfh cells and B6 B cells tested for total IgG (**W**) and IFN-γ (**X**). Ctrl corresponds to B cells alone. B cells from 3 B6 mice were each cultured with Tfh cells from 3 *Sle1b* or *Sle1b* *Gls1* CD4-KO mice. Each datapoint corresponds to an average of 3. Mean ± SEM, compared with Mann-Whitney *U* tests (**B**–**D**, **W**, and **X**), Dunnett’s or Šídák’s multiple-comparison tests. *P < 0.05; **P < 0.01; ***P < 0.001; ****P < 0.0001.

We next investigated the intrinsic role of glutaminolysis in CD4^+^ T cells in lupus with the *Gls1^fl/fl^* CD4-Cre system (*Gls1* CD4-KO), which has been previously used to investigate glutaminolysis in Th1 and Th17 cells (3). In addition to the B6 background, we bred *Gls1* CD4-KO to the *Sle1b* lupus-susceptibility locus, yielding 4 groups of mice: *Gls1* CD4-KO and *Gls1^fl/fl^* (“B6”) controls, and *Sle1b Gls1* CD4-KO with *Sle1b* controls. We first compared the 4 groups at 9–12 months of age, when *Sle1b* mice show a full expression of autoimmune phenotypes, including the production of anti-dsDNA IgG (31). *Sle1b Gls1* CD4-KO mice showed a reduced number of splenocytes as well as a reduced production of anti-dsDNA IgG as compared with *Sle1b* controls, while there was no difference between B6 *Gls1* CD4-KO and B6 mice (Figure 5, A and B). *Sle1b* mice presented spontaneous large splenic GCs that contain numerous CD4^+^ T cells. *Gls1* CD4-KO reduced the size of *Sle1b* GCs as well as the number of CD4^+^ T cells they contain (Figure 5, C and D), which could be a direct consequence of the reduced GC size, as well as an impaired ability of Tfh cell localization.

*Sle1b* CD4^+^ T cells showed a higher OXPHOS and glycolysis than B6 CD4^+^ T cells (Supplemental Figure 4, A and B). *Gls1* CD4-KO did not change these parameters in B6, but it decreased the maximal OXPHOS and glycolysis in *Sle1b* CD4^+^ T cells, suggesting a requirement for glutaminolysis under metabolic stress in these T cells. There was a trend for *Sle1b* CD4^+^ T cells for being more glycolytic than B6, which was accentuated by *Gls1* CD4-KO (Supplemental Figure 4C). *Gls1* CD4-KO decreased the frequency of *Sle1b* CD4^+^ T cells as well as their activation, evaluated by CD69 expression and Tem cell frequency (Figure 5, E–G). A similar trend was observed on the B6 background, but it was significant only for the frequency of Tem cells. The frequency of Tfh cells was similar between groups, but *Sle1b* mice presented a higher frequency of Texfh cells that was reduced by *Gls1* CD4-KO (Figure 5, H and I). The frequency of Tfr and Tfexfr cells was similar between groups (Figure 5, J and K). However, both Tfh/Tfr and Texfh/Texfr ratios were higher in *Sle1b* than in B6 mice, and the Texfh/Texfr ratio was reduced by *Gls1* CD4-KO in *Sle1b* mice (Figure 5, L and M). As in W.Yaa mice treated with DON (Figure 2, I and J, and Supplemental Figure 3, A–D), *Gls1* CD4-KO reduced ICOS expression not only in Tfh-related cells (Figure 5, N–Q), but also in Tn cells and nTregs in *Sle1b* mice (Supplemental Figure 5A). The reduction of ICOS expression by *Gls1* CD4-KO in B6 mice was less consistent, occurring only in Tfh, Tfr, and Tn cells (Figure 5, N, P, and S, and Supplemental Figure 5A). Finally, confirming the results obtained in W.Yaa mice (Figure 2, M and N), *Sle1b* Tfh and Texfh cells expressed higher levels of PD-1 than B6 counterparts, but it was not changed by the inhibition of glutaminolysis (Supplemental Figure 5B).

The abundance of amino acids, including glutamine, regulates cellular metabolism through the mTOR and GCN2 pathways in opposite directions (44, 45). *Gls1* CD4-KO T cells accumulate unprocessed glutamine (Figure 4C), which could activate mTOR. It has, however, no effect on the expression of the major targets of mTORc1, mTORc2, or GCN2 in Tfh and Texfh cells from either B6 or *Sle1b* mice (Supplemental Figure 5, C–F), therefore eliminating these pathways as a mechanistic link between the inhibition of glutaminolysis and the observed phenotypes in these T cell subsets.

*Sle1b* mice have higher frequencies of B cells and GC B cells that were not changed by *Gls1* CD4-KO (Figure 5, R and S). Their high DZ/LZ GC B ratio and frequency of ABCs were, however, reduced by *Gls1* CD4-KO (Figure 5, T and U). Finally, in contrast to DON-treated W.Yaa mice, there was no effect of *Gls1* CD4-KO on *Sle1b* plasma cells, possibly because their frequency was similar between *Sle1b* and B6 mice (Figure 5V). We next evaluated the functional effect of *Gls1* CD4-KO on the ability of *Sle1b* Tfh cells to help B cells in cocultures. We used B6 B cells to focus on the Tfh phenotypes. *Gls1* CD4-KO Tfh cells induced a lower secretion of IgG and produced a lower amount of IFN-γ than Sle1b Tfh cells (Figure 5, W and X). These results suggest that *Gls1* CD4-KO functionally impaired *Sle1b* Tfh cells.

We evaluated the effect of *Gls1* CD4-KO in 2- to 3-month-old *Sle1b* mice before they produced autoantibodies and in age-matched B6 controls. *Gls1* CD4-KO reduced the frequency of CD4^+^ T cells in both strains (Supplemental Figure 6A) to a greater extent than in older mice (Figure 5E). The frequency of Tfh and Tfr cells as well as the Tfh/Tfr ratio were low, with no significant difference between strains with and without *Gls1* CD4-KO (Supplemental Figure 6, B–D). However, the frequency of Texfh and Texfr cells, as well as the Texfh/Texfr ratio, were already higher in *Sle1b* than in B6 mice (Supplemental Figure 6, E and F), and, as in older mice (Figure 5M), the latter was reduced by *Gls1* CD4-KO (Supplemental Figure 6G). ICOS expression was also already higher on *Sle1b* than B6 Tfh and Texfh cells, and it was downregulated by *Gls1* CD4-KO (Supplemental Figure 6, H and I). Finally, a higher proliferation in *Sle1b* Tn cells was reduced by *Gls1* CD4-KO (Supplemental Figure 6J). *Gls1* CD4-KO increased the frequency of *Sle1b* B cells (Supplemental Figure 6K). Young *Sle1b* mice presented a higher frequency of GC B cells in which the high DZ/LZ ratio was reduced by *Gls1* CD4-KO (Supplemental Figure 6, L and M). The frequency of ABCs and plasmablasts was also higher in young *Sle1b* mice than in B6 mice, with a trend for a reduction of the ABC frequency by *Gls1* CD4-KO (Supplemental Figure 6, N and O). Finally, there was no difference between groups for the low frequency splenic plasma cells (Supplemental Figure 6P).

Overall, these results showed that *Gls1* deficiency in *Sle1b* CD4^+^ T cells phenocopied most of the CD4^+^ T cell phenotypes induced by DON treatment in W.Yaa CD4^+^ T cells, with a most preeminent effect on ICOS expression and the Texfh/Texfr ratio, and with less consistent effect on B6 CD4^+^ T cells. Furthermore, this phenotype is associated with a functional impairment of Tfh cells. These results demonstrate that these phenotypes were targeted by both pharmacological and genetic inhibition of glutaminolysis in a cell-intrinsic manner, in 2 different lupus-prone genetic backgrounds. *Gls1* deficiency in *Sle1b* CD4^+^ T cells was also sufficient to reproduce the phenotypes obtained in B cells from W.Yaa mice treated with DON — i.e., the reduction of autoantibody production, DZ / LZ GC B cell ratio and the frequency of ABCs. These results suggest that these B cell phenotypes are driven in a cell-extrinsic manner by glutaminolysis in T cells.

### The inhibition of glutaminolysis in T cells reduced induced autoimmunity.

**Figure 6:**
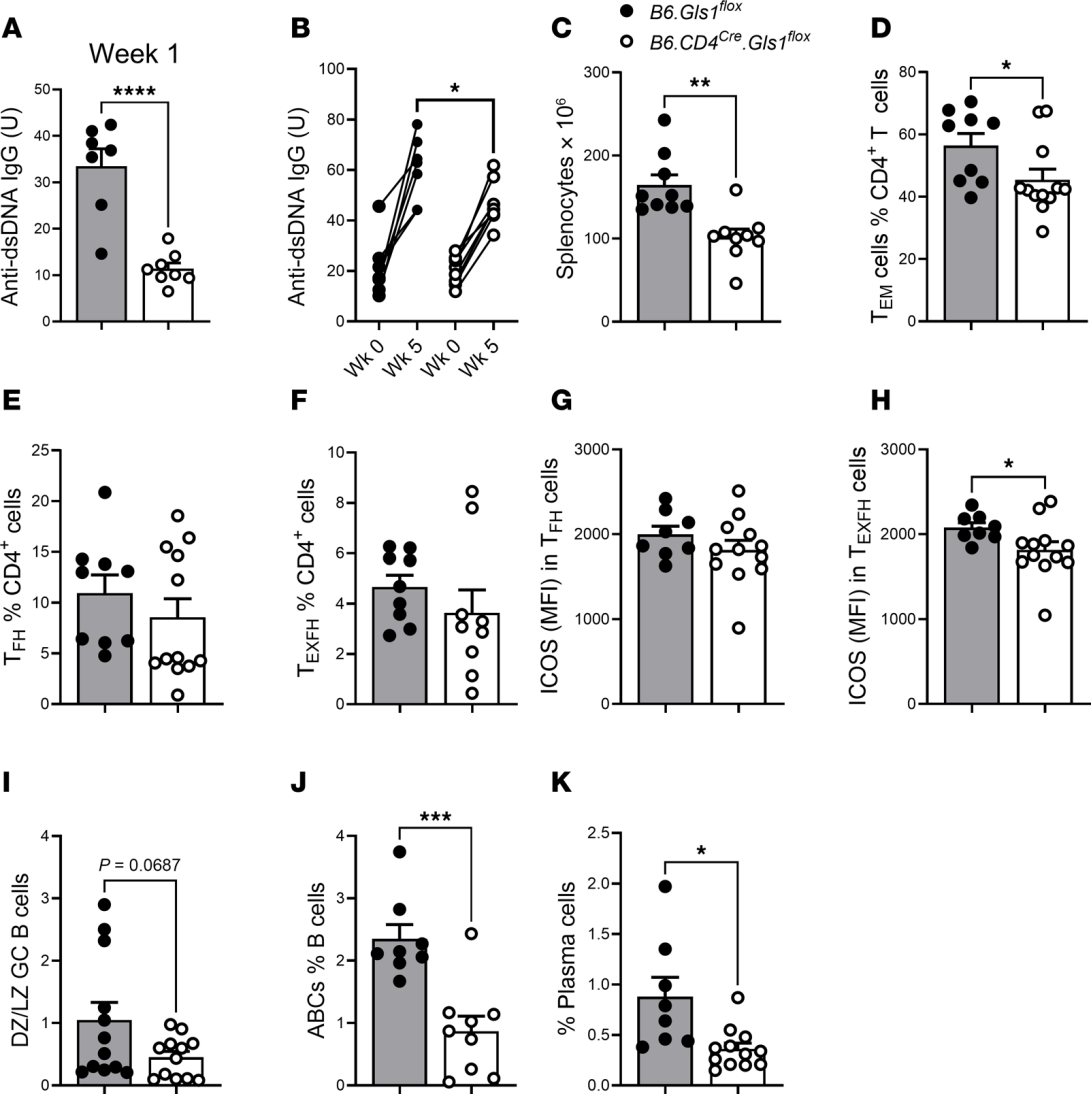
*Gls1* CD4-KO reduced the production of autoantibodies in B6 mice after cGVHD induction with selective effects on CD4^+^ T and B cells. (**A**) Serum anti-dsDNA IgG 1 week after induction. (**B**) Anti-dsDNA IgG before and 5 weeks after induction. Each pair represents 1 mouse. (**C**–**K**) Phenotypes evaluated 5 weeks after cGVHD induction. (**C**) Splenocyte numbers. Frequency of Tem (**D**), Tfh (**E**), and Texfh (**F**) cells. ICOS expression on Tfh (**G**) and Texfh (**H**) cells. (**I**) DZ/LZ GC B cell ratio. Frequency of ABCs (**J**) and plasma cells (**K**). *n* = 9 from 2 independent cohorts. Mean ± SEM, compared with *t* tests. *P < 0.05; **P < 0.01; ***P < 0.001; ****P < 0.0001.

To further investigate the role of *Gls1* deficiency in T cells in lupus-like autoimmunity, we used the chronic graft versus host disease model (cGVHD), in which an autoimmune response is induced in B6 mice by the transfer of H2-b alloreactive splenocytes from the B6.bm12 strain (46). This model largely depends on the extrafollicular activation of autoimmune lymphocytes in a CD4^+^ T cell–dependent manner (47). One week after induction, *Gls1* CD4-KO mice produced a reduced level of anti-dsDNA IgG as compared with B6 mice (Figure 6A). Although the anti-dsDNA IgG production increased in the subsequent weeks in *Gls1* CD4-KO mice, it was still lower than in B6 controls at week 5 (Figure 6B). *Gls1* CD4-KO did not have any effect on OXPHOS in the CD4^+^ T cells from these mice, but it increased glycolysis, resulting in a skewed basal OCR/ECAR ratio (Supplemental Figure 4, D–F). *Gls1* CD4-KO reduced the number of splenocytes and the frequency of Tem cells (Figure 6, C and D). As in *Sle1b*, the frequency of Tfh and Texfh cells was not affected with *Gls1* CD4-KO in cGVHD B6 mice (Figure 6, E–H), but ICOS expression was reduced in Texfh cells (Figure 6, I and J). Also, as with *Sle1b* mice, the frequency of GC B cells was unchanged, but there was a trend for a reduced DZ/LZ ratio (Figure 6I), and more importantly, there was a significantly reduced frequency of ABC and plasma cells (Figure 6, J and K). These results showed that *Gls1* CD4-KO induced similar cell-intrinsic effects in CD4^+^ T cells and extrinsic effects in B cells as observed in the *Sle1b* spontaneous model of autoimmunity, resulting in a reduction of autoantibody production in the cGVHD-induced model of lupus.

### The inhibition of glutaminolysis in T cells altered the T-dependent humoral response.

**Figure 7:**
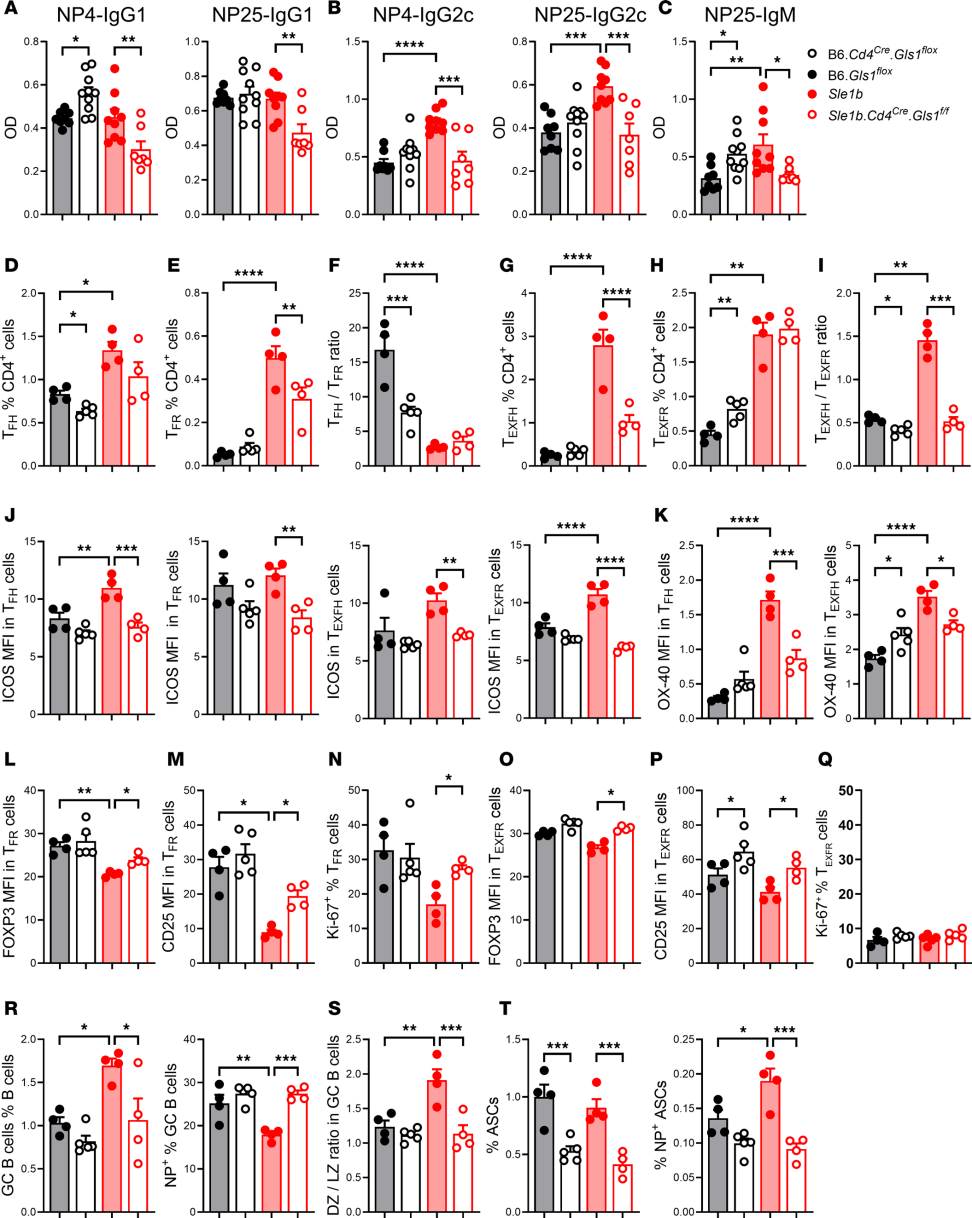
*Gls1* CD4-KO altered the humoral response to NP-KLH immunization. (**A**–**C**) Mice were immunized with NP-KLH and serum anti-NP4 and anti-NP25 IgG1 (**A**), IgG2c (**B**), and anti-NP25 IgM (**C**) were evaluated 1 week later. (**D**–**T**) Splenocyte analysis. Frequency of Tfh cells (**D**), Tfr cells (**E**), and Tfh/Tfr ratio (**F**). Frequency of Texfh cells (**G**), Texfr cells (**H**), and Texfh/Texfr ratio (**I**). (**J**) ICOS expression on Tfh, Tfr, Texfh, and Texfr cells. (**K**) OX-40 expression on Tfh and Texfh cells. Expression of FOXP3 (**L**) and CD25 (**M**) and frequency of proliferating Ki-67^+^ cells (**N**) in Tfr cells. Expression of FOXP3 (**O**) and CD25 (**P**) and frequency of proliferating Ki-67^+^ cells (**Q**) in Texfr cells. (**R**) Frequency of total GC B cells and NP^+^ in GC B cells. (**S**) DZ/LZ ratio in GC B cells. (**T**) Frequency of total and NP^+^ ASCs. All MFI values are shown as 1 × 10^–2^ of the obtained values. Means + SEM, *n* = 4–10 compared with Šídák’s or Dunnett’s (**O**) multiple-comparison tests. *P < 0.05; **P < 0.01; ***P < 0.001; ****P < 0.0001.

To further investigate the effect of *Gls1* CD4-KO on T cell help to B cells, we evaluated the primary response to a protein antigen, NP-KLH in alum, 7 days after immunization on both B6 and *Sle1b* backgrounds. *Gls1* CD4-KO increased the production of high-affinity anti-NP4 IgG1, the main isotype elicited by NP immunization, on the B6 background but decreased both high- and low-affinity anti-NP IgG1 on the *Sle1b* background (Figure 7A). Consequently, *Gls1* CD4-KO increased anti-NP IgG1 affinity, measured by the NP4/NP25 ratio, on the B6 but not the *Sle1b* background (Supplemental Figure 7A). We also measured anti-NP antibodies of the IgG2c isotype, which are elicited by IFN-γ–producing CD4^+^ T cells. *Sle1b* mice produced more anti-NP4 and NP25 IgG2c than B6 mice, and it was reduced to B6 levels by *Gls1* CD4-KO (Figure 7B). The affinity of anti-NP IgG2c was not affected by *Gls1* CD4-KO (Supplemental Figure 7A). The frequency of IFN-γ^+^CD4^+^ T cells was similar between immunized B6 and *Sle1b* mice, and it was increased by *Gls1* CD4-KO on the B6 background. However, the absolute number of IFN-γ^+^CD4^+^ T cells doubled in *Sle1b* mice as compared with B6, and it was greatly reduced by *Gls1* CD4-KO (Supplemental Figure 7B). These results are consistent with the amount of anti-NP IgG2c antibodies produced by *Sle1b* and *Sle1b Gls1* CD4-KO immunized mice. Finally, immunized *Sle1b* mice produced more anti-NP25 IgM than B6 controls and, as for anti-NP4 IgG1, *Gls1* CD4-KO had opposite effects on the 2 backgrounds; it increased in B6 and decreased in *Sle1b* (Figure 7C).

Immunized *Sle1b* mice presented a higher frequency of Tfh cells than B6 controls, which was decreased by *Gls1* CD4-KO on the B6 but not *Sle1b* background (Figure 7D). *Gls1* CD4-KO decreased the frequency of Tfr cells on the *Sle1b* but not B6 background, leading to a decreased Tfh/Tfr ratio on the B6 background (Figure 7, E and F). Immunized *Sle1b* mice developed a high frequency of Texfh cells that was clearly decreased by *Gls1* CD4-KO, while the high frequency of Texfr cells was unchanged, leading to a decreased Texfh/Texfr ratio (Figure 7, G–I). As observed in unmanipulated mice, *Gls1* CD4-KO decreased ICOS expression on *Sle1b* Tfh, Tfr, Texfh, and Texfr cells (Figure 7J). *Gls1* CD4-KO also decreased the expression of OX-40 on *Sle1b* Tfh and Texfh cells, where it was higher than on the B6 background (Figure 7K). On the other hand, *Gls1* CD4-KO increased FOXP3 and CD25 expression in Tfr cells as well as their proliferation on the *Sle1b* background (Figure 7, L–N). *Gls1* CD4-KO also increased FOXP3 and CD25 expression in Texfr cells on both backgrounds, but it had no effect on their proliferation, which was low (Figure 7, O–Q). These results suggest that *Gls1* CD4-KO negatively affected Tfh and Texfh cells while promoting Tfr and Texfr cells, especially on the *Sle1b* background. In B cells, *Gls1* CD4-KO reduced the elevated frequency of total *Sle1b* GC B cells but, surprisingly, increased the frequency of *Sle1b* NP^+^ GC B cells, which was lower than on the B6 background (Figure 7R). *Gls1* CD4-KO also reduced the DZ/LZ ratio (Figure 7S). Finally, *Gls1* CD4-KO reduced the frequency of total as well as NP^+^ ASCs on both backgrounds (Figure 7T).

We also assessed the recall response to NP-KLH in mice boosted 6 weeks after primary immunization. As with ex vivo and cGVHD CD4^+^ T cells, *Gls1* CD4-KO skewed the basal OCR/ECAR ratio in the CD4^+^ T cells of immunized mice in favor of glycolysis (Supplemental Figure 4, G–I). *Gls1* CD4-KO decreased frequency of Tfh cells and the Tfh/Tfr ratio in *Sle1b* mice (Supplemental Figure 7C). ICOS expression was decreased by *Gls1* CD4-KO in Tn cells from both backgrounds, while there was no change in Tfh cells (Supplemental Figure 7D). This suggests that *Gls1* CD4-KO may limit the initiation of the Tfh program in Tn cells. At this time point, the B cell phenotypes were similar between groups without significant effect of *Gls1* CD4-KO (data not shown).

Overall, these results show that *Gls1* CD4-KO affects the antibody response to immunization with a protein antigen, supporting the hypothesis that autoimmune and healthy CD4^+^ T cells use glutaminolysis in a different manner to help B cells.

### The inhibition of glutaminolysis in T cells altered the response of autoimmune mice to LCMV infection.

**Figure 8:**
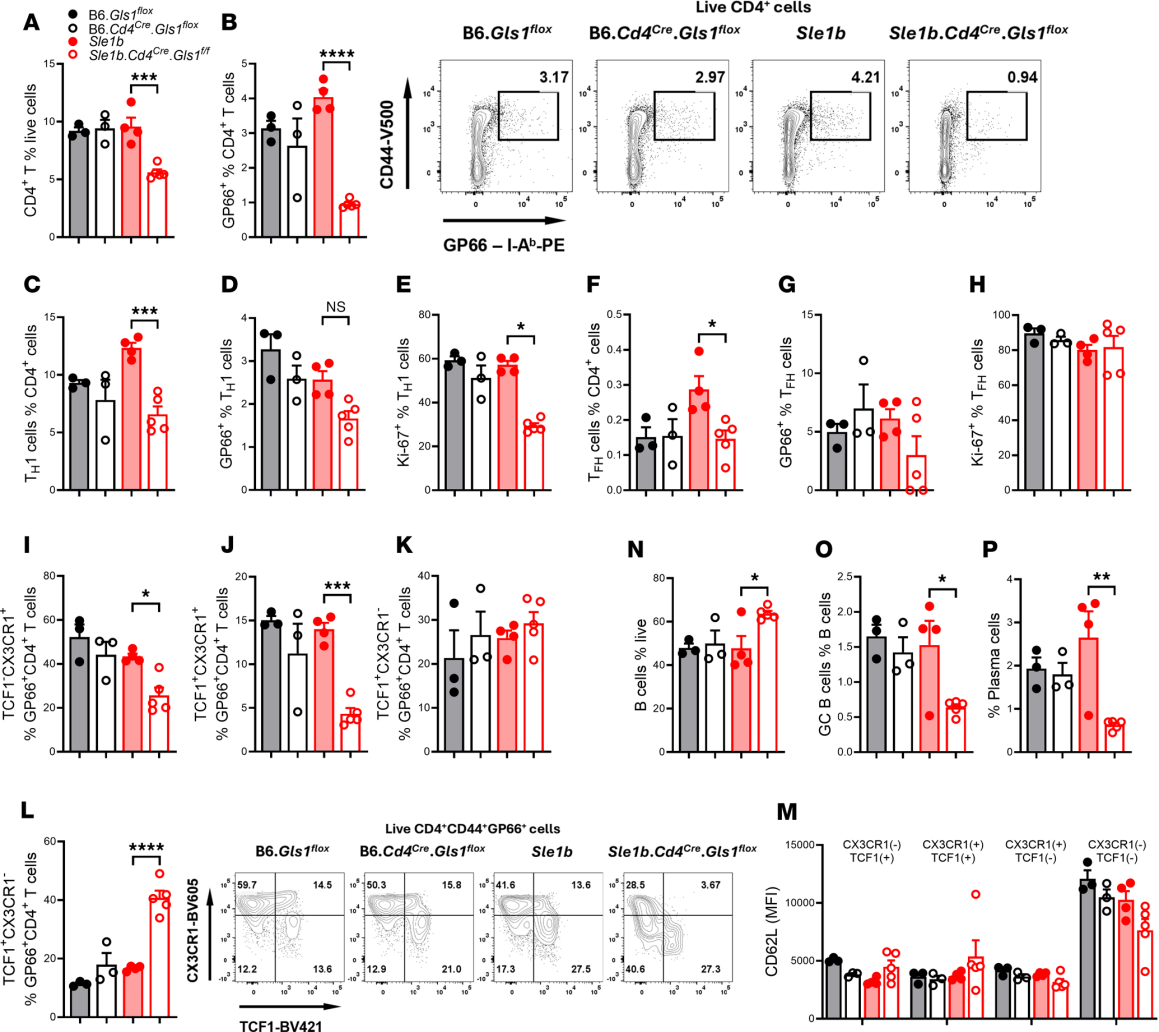
*Gls1* CD4-KO reduced CD4^+^ T and B cell responses to LCMV infection. Splenic CD4^+^ T cells and B cells were analyzed 7 days after infection with LCMV Armstrong. (**A** and **B**) Frequency of total (**A**) and GP66^+^ (**B**) CD4^+^ T cells, with representative FACS plots on the right. (**C**–**E**) Frequency of total (**C**), GP66^+^ (**D**), and Ki-67^+^ Th1 cells (**E**). (**F**–**H**) Frequency of total (**F**), GP66^+^ (**G**), and Ki-67^+^ (**H**) Tfh cells. (**I**–**L**) Frequency of TCF1^–^CX3CR1^+^ (**I**), TCF1^+^CX3CR1^+^ (**J**), TCF1^+^CX3CR1^–^ (**K**), and TCF1^+^CX3CR1^–^ (**L**) GP66^+^ CD4^+^ T cells, with representative FACS plots on the right. (**M**) CD62L expression in the four GP66^+^ CD4^+^ T cell subsets. (**N**–**P**) Frequency of B cells (**N**), GC B cells (**O**), and plasma cells (**P**). Mean ± SEM, *n* = 3–5 compared with Šídák’s multiple or Dunnett’s (**E** and **P**) comparisons tests. *P < 0.05; **P < 0.01; ***P < 0.001; ****P < 0.0001.

To specifically determine the effect of *Gls1* CD4-KO on antigen-specific CD4^+^ T cells, we infected mice with the Armstrong strain of the lymphocytic choriomeningitis virus (LCMV) and evaluated the phenotypes of splenic total CD4^+^ T cells as well as LCMV-specific GP66^+^CD4^+^ T cells 7 days later. *Gls1* CD4-KO reduced the frequency of total and GP66^+^CD4^+^ T cells in *Sle1b* mice (Figure 8, A and B). The same result was obtained for CD44^+^PD-1^–^PSGL1^+^CD4^+^ T cells, corresponding to Th1 cells (48), and *Gls1* CD4-KO reduced their proliferation (Figure 8, C–E). The frequency of total Tfh cells was also reduced by *Gls1* CD4-KO in *Sle1b* mice but not that of GP66^+^ cells Tfh cells or the proliferation of Tfh cells (Figure 8, F–H). We further analyzed GP66^+^CD4^+^ T cells according to their expression of TCF1, a transcription factor intrinsically required for the differentiation of viral-specific Tfh cells (49), and CX3CR1, a maker of terminally differentiated Th1 cells in viral infections (50). CX3CR1 is also a marker of IFN-γ–producing Tfh1 cells (51), a subset of Tfh cells that is expanded in some patients with SLE and mouse models of the disease (52–54). The frequency of the TCF1^–^ CX3CR1^+^ and, to a greater extent, TCF1^+^CX3CR1^+^ subsets of GP66^+^ CD4^+^ T cells were decreased by *Gls1* CD4-KO in *Sle1b* mice, which was compensated by an increase in the double-negative subset, while there was no difference for the TCF1^+^CX3CR1^–^ subset (Figure 8, I–M). TCF1^–^CX3CR1^–^ T cells are poorly defined. Their level of GP66 expression was similar to that of the other subsets; they expressed low level of CD162, consistent with being CX3CR1^–^, and they do not express Tfh markers (data not shown). They expressed however high levels of CD62L (Figure 8M). These TCF1^–^CD62L^hi^ cells may correspond to uncommitted transitional effector cells. Their increased frequency in *Sle1b* GLS1 CD4-KO GP66^+^ T cells supports that glutaminolysis inhibition impaired the differentiation of virus-specific effector T cells on the autoreactive genetic background, with a greater effect on Tfh1 cells. *Gls1* CD4-KO increased the frequency of B cells in *Sle1b*-infected mice (Figure 8N) and decreased the frequency of GC B cells and plasma cells (Figure 8, O and P), also suggesting a block in effector B cell differentiation. Overall, these results suggest that autoimmune CD4^+^ T cells have a higher requirement for glutaminolysis to respond to LCMV infection as compared with healthy controls. This requirement affects specifically Th1 and Tfh1 cells. Consequently, infected *Sle1b* *Gls1* CD4-KO mice develop a reduced GC B cell and plasma cell expansion.

## Discussion

This study focused on the role of glutaminolysis in Tfh cells in lupus-prone mice as compared with healthy controls. Tfh cells are a subset of CD4^+^ T cells that are critical for the production of autoantibodies in SLE (55), but the role of glutaminolysis has been relatively less studied in Tfh cells comparatively to Th1 and Th17 cells (3, 4, 6). We have previously shown that the pharmacological inhibition of glutaminolysis with DON reduced the production of anti-dsDNA IgG antibody in TC lupus-prone mice. DON did not change the frequency of TC Tfh cells, but it reduced their ICOS and BCL-6 expression (16). The beneficial effect of glutaminolysis inhibition on autoantibody production was confirmed in another lupus-prone MRL/lpr model treated with CB839, along with a modest reduction of Tfh cell frequency (5). We have shown that glutaminolysis is not required for Tfh cell differentiation from purified CD4^+^ T cells from either lupus or control mice (18). However, the increased glutamate metabolism and differential expression of glutamine transporters on TC Tfh cells as compared with healthy controls (19) suggested that glutamine contributes to the expansion of lupus Tfh cells and/or their enhanced functions.

Here, we used the TLR7-driven W.Yaa model to further investigate the contribution of glutaminolysis to lupus Tfh cells comparatively to healthy controls. We confirmed that the pharmacological inhibition of GLS1 reduced autoantibody production, although to a relative modest level as compared with the inhibition of glycolysis in the same strain (25). The DON treatment, however, reduced immune complex-induced renal pathology. At the cellular level, we confirmed that GLS1 inhibition reduced the frequency of Th17 cells but not that of Th1 cells (3), and it had no effect on the frequency of Tregs, contrary to results obtained with in vitro polarization (56). DON did not reduce the frequency of W.Yaa Tfh and Texfh cells. However, it reduced their expression of ICOS and OX-40, which are critical regulators of Tfh cell differentiation, maintenance, and function (26, 27). The downregulation of ICOS was observed across all CD4^+^ T cell subsets, including naive T cells, suggesting that DON may impair their differentiation into Tfh cells. A reduced ICOS expression may also functionally impair Tfh cells. DON-treated and untreated W.Yaa mice presented similar frequency of GC B cells, but DON-treated mice presented a relatively lower number of GC B cells in the dark zone, where they migrate after receiving a survival signal from Tfh cells in the light zone, proliferate, and undergo somatic hypermutation before differentiating into plasma cells (57). Consistent with this observation, DON treatment also reduced the frequency of plasma cells. The increased TLR7 signaling in W.Yaa mice has been shown by others to expand the GC dark zone (58, 59). In addition, the related *Sle1.Yaa* model showed an altered GC B cell dynamics associated with an impaired CD40 signaling in LZ B cells and correlated with plasma cell accumulation (60). Our results support the hypothesis that DON either counteracted excessive TLR7 signaling in B cells or altered the function of Tfh cells to restore GC B cell dynamics and their differentiation into plasma cells. DON also reduced the frequency of ABCs, a population greatly expanded in W.Yaa mice. It could be due to a direct effect of DON though the inhibition of glycolysis, since ABCs are highly glycolytic (61), or to the reduced Texfh/Texfr ratio and the reduced expression of ICOS on Texfh cells, both functionally reducing their help to ABCs.

DON uniquely modulated the transcriptome and metabolome of W.Yaa Tfh cells. In addition to the expected downregulation of glutamine metabolism, DON altered the metabolism of other amino acids. DON also reduced the expression of cell cycle genes as well as genes in the type I IFN pathway, supporting the hypothesis that the TRL7/type I IFN pathway is sustained by glutaminolysis in W.Yaa Tfh cells. DON had little effect on the transcriptome and metabolome of Tfh cells in B6 mice, which was corroborated by minor phenotypic changes, suggesting a unique requirement of lupus Tfh cells for glutaminolysis. As reported by others (3, 6), we confirmed that the inhibition of glutaminolysis reduced glycolysis in CD4^+^ T cells, but here we showed it affected specifically lupus Tfh cells with little effect in healthy Tfh cells. The inhibition of glutaminolysis with DON and the inhibition of glycolysis with 2DG (22, 25) had however distinctive effects on W.Yaa Tfh cells, the most striking being that only 2DG decreased their frequency. In addition, only DON reduced N-glycosylation in W.Yaa Tfh cells. 2DG has been reported to reduce N-glycosylation in cancer cells (62), but there is no evidence that it also occurs in T cells. More detailed metabolic studies will be necessary to dissect the intersection of glycolysis and glutaminolysis in lupus T cells, including N-glycosylation.

To better understand the intrinsic role of glutaminolysis in lupus CD4^+^ T cells, we analyzed the effect of *Gls1* deficiency in T cells on the autoimmune phenotypes of mice carrying the lupus-susceptibility locus *Sle1b*, which drives the production of autoantibodies associated with an expansion of Tfh cells and GC B cells (28, 33). We showed that *Gls1* deficiency in T cells was sufficient to reduce autoantibody production and to phenocopy the effect of DON treatment on Tfh and B cells. This included the reduction of ICOS expression, the Texfh/Texfr and the GC DZ/LZ ratios, as well as the frequency of ABCs. Using this model, we showed that *Gls1*-deficient *Sle1b* Tfh cells were defective in inducing the production of class-switched antibodies from cocultured B cells. These results were largely confirmed with the cGVHD-induced model of lupus on the B6 background. These results strongly suggest that lupus Tfh cells use glutaminolysis to enhance their help to B cells supporting autoantibody production, in association with altered GC B cell dynamics and an expansion of the ABC subset. We further show that the inhibition of glutaminolysis in T cells affected T-dependent humoral responses as well as responses to an acute viral infection in a different manner between an autoimmune and a healthy genetic background. These results support the hypothesis that glutaminolysis supports the function of lupus Tfh cells.

Mechanistically, glutaminolysis may support lupus Tfh cells by promoting the expression of costimulatory molecules, especially ICOS. Since ICOS signaling is required for the differentiation and maintenance of Tfh cells by inducing *Bcl6* expression (26), a higher ICOS expression contributes to Tfh cell maintenance and functions. A limitation of our study is that we have not yet identified the molecular mechanism linking glutaminolysis to ICOS and OX-40 expression. Since the expression of other Tfh effector molecules, such as PD-1, was not reduced by either pharmacological or genetic inhibition of GLS1, it suggests a gene-specific rather than a global effect. TCR activation triggers *Icos* transcription (34). W.Yaa Tfh cells express higher levels of *Icos* than B6 Tfh cells, consistent with a stronger TCR signaling (22). It has been recently shown that *Icos* expression is regulated epigenetically by DNA methylation (63), a process at least partially regulated by the glutaminolysis metabolite αKG. It will be, therefore, of great interest to assess DNA methylation at the *Icos* promoter in lupus T cells relative to glutaminolysis. Additional posttranscriptional regulation of ICOS expression that are likely to also be linked to glutaminolysis will need to be investigated. The Gls1-CD4-Cre model that we have developed will be essential to conduct these experiments and compare the autoimmune to control backgrounds.

Overall, these results have identified a cell-intrinsic role for glutaminolysis in Tfh cells expanded in multiple models on lupus, which indirectly affects B cell differentiation and antibody production. The glutaminase inhibitor CB839 and a DON-derivative prodrug are in clinical trials in oncology with promising results (64, 65). Our study on Tfh cells combined with other studies on Th17 cells (3, 6) in preclinical models suggest that these treatments may be beneficial to treat patients with SLE. A limitation of the study, however, is that we do not know the role of glutamine metabolism in the expansion of pathogenic T cells in patients with SLE, including in Tfh cells, at least partially due to the limitations of in vitro polarization. Glutaminolysis is a promising target in cancer patients, in which inhibition of glutamine transporters or degrading enzymes is being investigated in multiple clinical trials (66). It would, therefore, be highly beneficial to validate experimental approaches in which the role of glutamine in the T cells could be evaluated in patients with SLE.

## Methods

### Sex as a biological variable.

Two models of autoimmunity were used in this study with corresponding sex-matched controls: The W.Yaa model used only males and the *Sle1b* model with *Gls1* CD4-KO used only females.

### Mice and treatment.

NZW/J (JAX:001058) females and BXSB/MpJ (JAX:000740) males were bred to produce F1 male progeny (W.Yaa) used in this study. C57BL6/J (B6, JAX:000664) were bred at the UTHSCSA animal facility. Twelve-week-old W.Yaa and B6 males were treated with intra-peritoneal injections of DON (Sigma-Aldrich, 1.6 mg/kg) in PBS or PBS control 3 times a week for 2 or 4 weeks.

B6.*Gsl1fl/fl* mice (3) were bred to CD4-Cre mice (JAX:017336) as well as B6.*Sle1b* mice (31) to produce 4 strains B6.*Gsl1^fl/fl^* (B6), B6.*Gsl1^fl/fl^-CD4-Cre* (*Gls1* CD4-KO), B6.*Sle1b* (*Sle1b*), and B6.*Sle1b.Gsl1^fl/fl^-CD4-Cre* (*Sle1b* *Gls1* CD4-KO). Only females were used from these strains at either 2–3 months or 9–12 months of age.

cGVHD was induced as previously described (46). Briefly, B6 and *Gls1* CD4-KO hosts received 8 × 10^7^ splenocytes from B6.H-2bm12 mice (JAX #001162) via i.p. injection. Serum was collected weekly after induction and stored for ELISA measurement of autoantibodies. Hosts were sacrificed 5 weeks after transfer and splenocytes were analyzed by flow cytometry.

For dinitrophenyl-keyhole limpet hemocyanin (NP-KLH) immunization, 8- to 10-week-old mice received an i.p. injection of 100 μg NP(31)-KLH (Biosearch Technology) in alum as previously described (16) and were sacrificed 7 days later to evaluate splenic CD4^+^ T cell and B cell phenotypes. The recall response was evaluated in mice boosted with the same immunogen at week 6 and sacrificed at week 7. Some mice in the same age group were infected i.p. with 2 × 10^5^ PFU LCMV-Armstrong and sacrificed 7 days later for immunophenotyping of splenocytes. Mice were maintained in SPF conditions at the UTHSA.

### Antibody and cytokine measurements.

Anti-dsDNA IgG was detected by ELISA in sera diluted 1:100 as previously described (16). NP-specific antibodies were measured in the serum of NP-KLH immunized mice collected at weeks 1 or 7 after the primary immunization. ELISA plates were coated with NP(4)- or NP(25)-BSA (high or low affinity, respectively) (Biosearch Technology), followed by incubation with 1:5,000 (IgG1) or 1:1,000 (IgG2c) diluted serum samples, and developed with alkaline phosphatase-conjugated goat anti-mouse IgG1 (1071-04, 1:1000) or IgG2c (1081-04, 1:1000), both from Southern Biotech. To compare the effect of *Gls1* CD4-KO on *Sle1b* Tfh cells to provide help to B cells, Tfh cells sorted from *Sle1b* and *Gls1* CD4-KO mice were cocultured with purified B cells from B6 mice using B cell isolation kit (Miltenyi). Tfh cells (1.5 × 10^5^ cells/mL) were resuspended in cRPMI with B cells (2.5 × 10^5^ cells/mL) in the presence of anti-CD3 mAb (2 μg/mL) and F(ab’)_2_ anti-mouse IgM (5 μg/mL Jackson ImmunoResearch Laboratories). B cells alone were used as baseline control. Total IgG and IFN-γ were measured in undiluted supernatants collected 3.5 days later. IgG was measured by ELISA using plates coated with goat anti-mouse IgG (Accurate Chemical) at a concentration of 1 μg/mL in 0.1 M bicarbonate buffer and detected with alkaline phosphatase-conjugated goat anti-mouse IgG (Millipore/Sigma) diluted 1:1,000 and of PNPP substrate solution (Southern Biotech). Quantitation was performed relative to a serial dilution of standard IgG. IFN-γ was measured using the LEGEND MAX Mouse IFN-γ ELISA Kit according to the manufacturer’s instructions (BioLegend). All ELISA samples were run in duplicate.

### Flow cytometry and cell sorting.

Flow cytometry was performed on splenocyte suspensions prepared as previously described (16). Antibodies and stains used in this study are listed in Supplemental Table 1. Dead cells were excluded with fixable viability dye. For intracellular staining, cells were fixed and permeabilized using the FOXP3/Transcription Factor Staining Buffer (Thermo Fisher). Data were acquired using an LSRFortessa or FACSymphony A5, and they were analyzed with FlowJo V10 (BD Bioscience). Gating strategies for CD4^+^ T cell and B cell subsets are shown in Supplemental Figure 2. Tfh cells were gated as FOXP3^–^CD44^+^PD-1^+^CXCR5^+^BCL6^+^PSGL1^lo^CD4^+^ T cells, Tfr cells as FOXP3^+^PD-1^+^CXCR5^+^BCL6^+^PSGL1^lo^CD4^+^ T cells, Texfh cells as FOXP3^–^CD44^+^PD-1^+^CXCR5^–^BCL6^–^PSGL1^lo^CD4^+^ T cells, and Texfr cells as FOXP3^+^CD44^+^PD-1^+^CXCR5^–^BCL6^–^PSGL1^lo^CD4^+^ T cells. In the LCMV infection experiment, Th1 cells were gated as CD44^+^PD-1^–^PSGL1^+^CD4^+^ T cells. I-A(b) LCMV GP 66-77 DIYKGVYQFKSV (GP66) tetramers were obtained from the NIH Tetramer core facility. For RNASeq and metabolomic analyses, Tfh cells were sorted as CD4^+^CD44^+^PD-1^+^PSGL-1^lo^ cells with a FACSARIA III cytometer. Purity was > 95% (Supplemental Figure 8).

### Histology.

GC were visualized in frozen sections of spleen stained with anti-CD4 APC, anti-IgD-PE and anti-GL7-AF488 as previously described (16). Paraformaldehyde-fixed paraffin kidney sections were stained with periodic acid Schiff (PAS). The type and extent of renal lesions were evaluated using a modification of the International Society of Nephrology and Renal Pathology Society classification of lupus nephritis and the NIH activity and chronicity indices in a blind manner. Renal parenchymal components, including glomeruli, vessels, tubules, and interstitium, distributed throughout the renal sections were assessed. At least 25 glomeruli per kidney were examined for the presence of mesangial expansion, mesangial hypercellularity, crescents and glomerulosclerosis, tubular dilatation, and tubular casts were quantified on a scale of 1 to 4. Renal pathology scores were calculated as the sum of the 5 criteria. IgG2a and C3 immune complexes as well as T cell and macrophage infiltration were detected in frozen sections as previously described (25). Imaging and quantitation were performed with a Keyence microscope Model BZ-X810 using the image stitching module.

### Western blotting.

Total protein extracted from magnetic bead-purified CD4^+^CD44^+^CD62L^–^ T cells were quantified using the BCA method. Immunoblotting was performed according to a standard protocol with primary antibodies and HRP-conjugated anti-rabbit or anti-mouse IgG secondary antibodies. Signal was detected using Signal-Fire-Elite. Image J software was used for densitometric analysis. For repeated measurements of different proteins on the same membrane, PVDF stripping buffer was applied and the membrane was reblocked before reprobing.

### RNA-Seq.

RNA was isolated from Tfh cells sorted by FACS from W.Yaa and B6 mice treated with DON or untreated controls using a RNeasy Plus Micro kit (Qiagen). cDNA libraries were constructed using a SMART-Seq HT (Takara), as previously described (67). Paired-end sequence reads (2 × 150 bp) were aligned to the mouse reference genomes (GRCm38) using STAR v2.7.5c. Normalized counts, represented by transcripts per million (TPM), were generated using RSEM v1.3.3. The raw transcript counts were used as input to determine significantly expressed genes by DESeq2 based on the criteria (TPM > 1, FDR < 0.05, fold change > 1.5). GSEA was performed using DAVID (https://david.ncifcrf.gov/).

### Metabolomic analysis.

Mitochondrial stress assays were conducted on an XF96 extracellular flux analyzer on magnetic-bead purified CD44^+^ CD4^+^ T cells or CD4^+^ T cells, as indicated for each experiment, as previously described (14). Data were normalized using a coupled Biotek Cytation 1 imager. FACS-sorted Tfh cells (same samples used for RNA-Seq analysis) were processed for metabolomic analysis as described previously (68). Metabolic feature alignment and curation were performed. After normalization to total ion chromatogram, intensities were tested for group significance using unpaired, 2-tailed Student *t* test. Metabolites were identified by comparison to the metabolomic library of purified standards. Metabolic pathway analysis was performed using *Mummichog*. The pathways represented by at least 3 significant metabolites are presented.

### Statistics.

Statistical analyses were performed using GraphPad Prism 9.0 software. Unless otherwise stated, differences between groups were evaluated by 1-way ANOVA with correction for multiple testing in Dunnet’s (data normally distributed unequal variance), Šídák’s (data normally distributed equal variance) or Kruskal-Wallis (data not normally distributed) multiple-comparison tests, or with unpaired or paired 2-tailed *t* tests or Mann-Whitney test (2 groups with data normally or not distributed, respectively) as indicated in the figure legends. The corresponding nonparametric tests were used when the data distribution deviated from normality. Results were expressed as mean ± SEM. Statistical significance was set at *P* < 0.05.

### Study approval.

This study was carried out in accordance with the guidelines from the *Guide for the Care and Use of Laboratory Animals* (National Academies Press, 2011). All animal protocols were approved by the IACUC of UTHSA (IACUC 20220032AR).

### Data availability.

The transcriptomic raw FastQ files have been made publicly available under the NCBI BioProject accession no. PRJNA1300446. The metagenomic raw dataset is available from the corresponding author upon request. All data shown in graphs is available in the Supporting Data Values file.

## Author contributions

Conceptualization was contributed by SCC, MM, and LM. Methodology was contributed by SCC, NZ, and YG. Investigation was contributed by SCC, YG, MVJ, DJ, LTP, ACG, CM, and CL. Review and editing was contributed by MM and LM. Resources were contributed by JCR. Funding acquisition was contributed by MM and LM. Supervision was contributed by LM.

## Funding support

This work is the result of NIH funding and is subject to the NIH Public Access Policy. Through acceptance of this federal funding, the NIH has been given a right to make the work publicly available in PubMed Central.

- NIH R01 AI154630 to LM and MM.
- R37AI128901 to LM.
- South Texas Medical Scientist Training Program NIH NIGMS/T32GM113896 and T32GM145432 to MVJ.

## Supplementary Material

Supplemental data

Supporting data values

## Version 1 09/16/2025

In-Press Preview

## Version 2 11/10/2025

Electronic publication
