# Supplementary material for: Intrinsic T cell glutaminolysis promotes autoimmunity in lupus-prone mice
Source: JCI Insight. 2025 Sep 16;10(21):e192286. doi: 10.1172/jci.insight.192286 (PMC12643493; doi:10.1172/jci.insight.192286)
Supplement: Supplemental data [file jciinsight-10-192286-s072.pdf]

Supplementary Table 1: Antibodies and stains

Target Antigen	Conjugation	Clone	vendor	Dilution	RRID #
β-ACTIN		8H10D10	Cell Signaling	1:4000	AB_3700
ATF4		23GB3140	Thermo Fisher	1:100	AB_3249075
BCL-6	BV750	K112-91	BD Biosciences	1:50	AB_3146163
cCBL		3B12	InVitrogen	1:1000	AB_11153576
CBL-B		polyclonal	InVitrogen	1:1000	AB_2851949
CD3e		145-2C11	BD Biosciences	2 ug/ml	AB_394590
CD4	BUV395	GK1.5	BD Biosciences	1:100	AB_2738426
	BV750		BioLegend	1:100	AB_2734150
	AF700	RM4-5	Thermo Fisher	1:100	AB_494001
	APC			1:100	AB_469323
CD5	AF700	53-7.3	BioLegend	1:100	AB_2687002
CD11c	APC	N418	Thermo Fisher	1:100	AB_469346
CD16/CD32		2.4G2	BD Biosciences	1:500	AB_394656
CD19	BV786	1D3	BD Biosciences	1:100	AB_2738141
CD21	BV711	7E9	BioLegend	1:100	AB_2876440
CD23	BV510	B3B4	BioLegend	1:100	AB_2563705
CD25	BV421	PC61	BD Biosciences	1:100	AB_11153485
CD44	PE	IM7	BioLegend	1:100	AB_312959
	V500		BD Biosciences	1:100	AB_312959
CD62L	APC	MEL-14	BD Biosciences	1:100	AB_398533
CD69	PE-Cy7	H1.2F3	BioLegend	1:100	AB_493564
CD86	BV650	GL-1	BioLegend	1:100	AB_11126147
CD93	BV750	AA4.1	BD Biosciences	1:100	AB_2871654
CD95 (Fas)	Biotin	Jo2	BD Biosciences	1:100	AB_395328
CD134 (OX-40)	BV650	OX-86	BD Biosciences	1:100	AB_2740249
CD138	BV605	281-2	BioLegend	1:100	AB_2562336
CD162 (PSGL-1)	BV605	2PH1	BD Biosciences	1:100	AB_2740114
	AF647		BD Biosciences	1:100	AB_2737807
CD184 (CXCR4)	BUV395	2B11	BD Biosciences	1:100	AB_2740007
CD185 (CXCR5)		2G8	BD Biosciences	1:100	AB_394302
CD278 (ICOS)	PE	7E.17G9	BD Biosciences	1:100	AB_394349
CD279 (PD-1)	eFluor 450	RMP1-30	Thermo Fisher	1:100	AB_11150068
	BV785	J4329F.1A12	BioLegend	1:100	AB_2563680
CX3CR1	BV605	SAO11F11	BioLegend	1:100	AB_2565999
FOXP3	FITC	FJK-16S	Thermo Fisher	1:100	AB_465243
GL7	AF450	GL7	Thermo Fisher	1:100	AB_10870775
	AF488			1:50	AB_2016717
GP66 tetramer	APC		NIH	1:15	
Helios	AF700	22F6	Thermo Fisher	1:100	AB_2784773
IgM F(ab')2		polyclonal	Thermo Fisher	5 ug/ml	AB_2338469
IgD	FITC	11-26c.2a	BD Biosciences	1:100	AB_394859
	PE			1:50	AB_647211
IL-10	PE-Cy7	JES5-16E3	BioLegend	1:100	AB_11150582
IL-17A	PE	TC11-18h10.1	BioLegend	1:100	AB_315464
IFN-γ	BV421	XMG1.2	BioLegend	1:100	AB_893526
Ki-67	BV711	SolA15	BD Biosciences	1:100	AB_2738406
NP	PE		Biosearch Tech	1:100	
p4E-BP1	AF647	236B4	Cell Signaling	1:100	AB_2097838
pAKT	PE/Cy7	SDRNR	Thermo Fisher	1:100	AB_2688172
pS6	Pacific Blue	D57.2.2E	Cell Signaling	1:100	AB_2797646
T-bet	PE-Cy7	eBio4B10	Thermo Fisher	1:100	AB_2784726

TCF1	BV421	S33-966	BD Biosciences	1:100	AB_2869822
Streptavidin	PerCP		BD Biosciences	1:100	

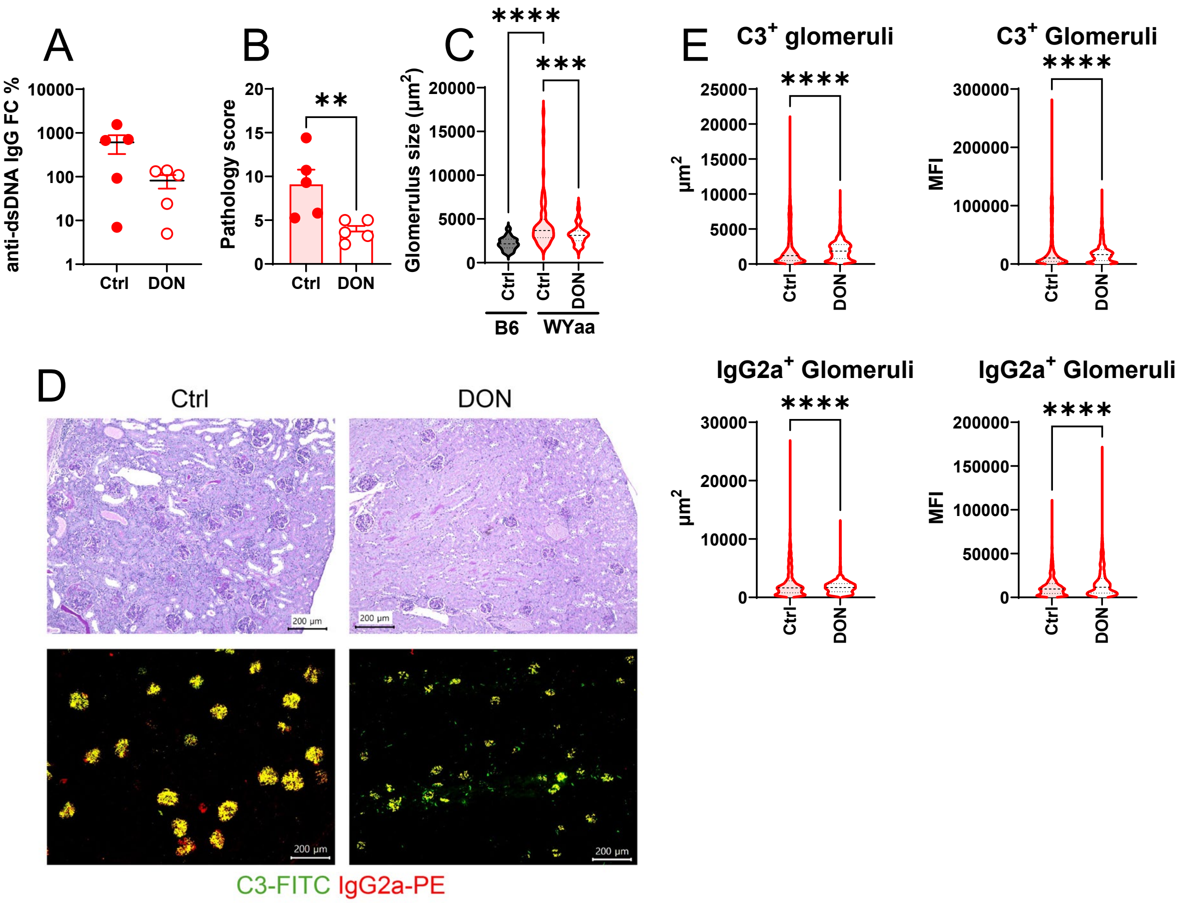

Supplemental Figure 1. DON treatment reduced renal pathology in W.Yaa mice. W.Yaa mice were treated with DON for 4 weeks, then compared to aged-matched W.Yaa and B6 controls (N = 5 each). A. Serum anti-dsDNA fold change between initial and terminal time points. B – C. Renal pathology scores (B), glomerulus size (computed on the total number of glomeruli in each sample N = 34 – 65, C) and representative images of PAS-stained kidney sections (10 X, D upper row). E. Quantification of C3 and IgG2a glomerular staining showing for each the size and MFI of total glomeruli positive for each antibody in entire longitudinal sections. Representative images of the overlay are shown in D lower row. Means + SEM, compared with t test (B). Medians and distributions compared by Dunnett's multiple comparisons tests (C) or Kolmogorov-Smirnov tests (E). **: P < 0.01; ***: P < 0.001; ****: P < 0.0001.

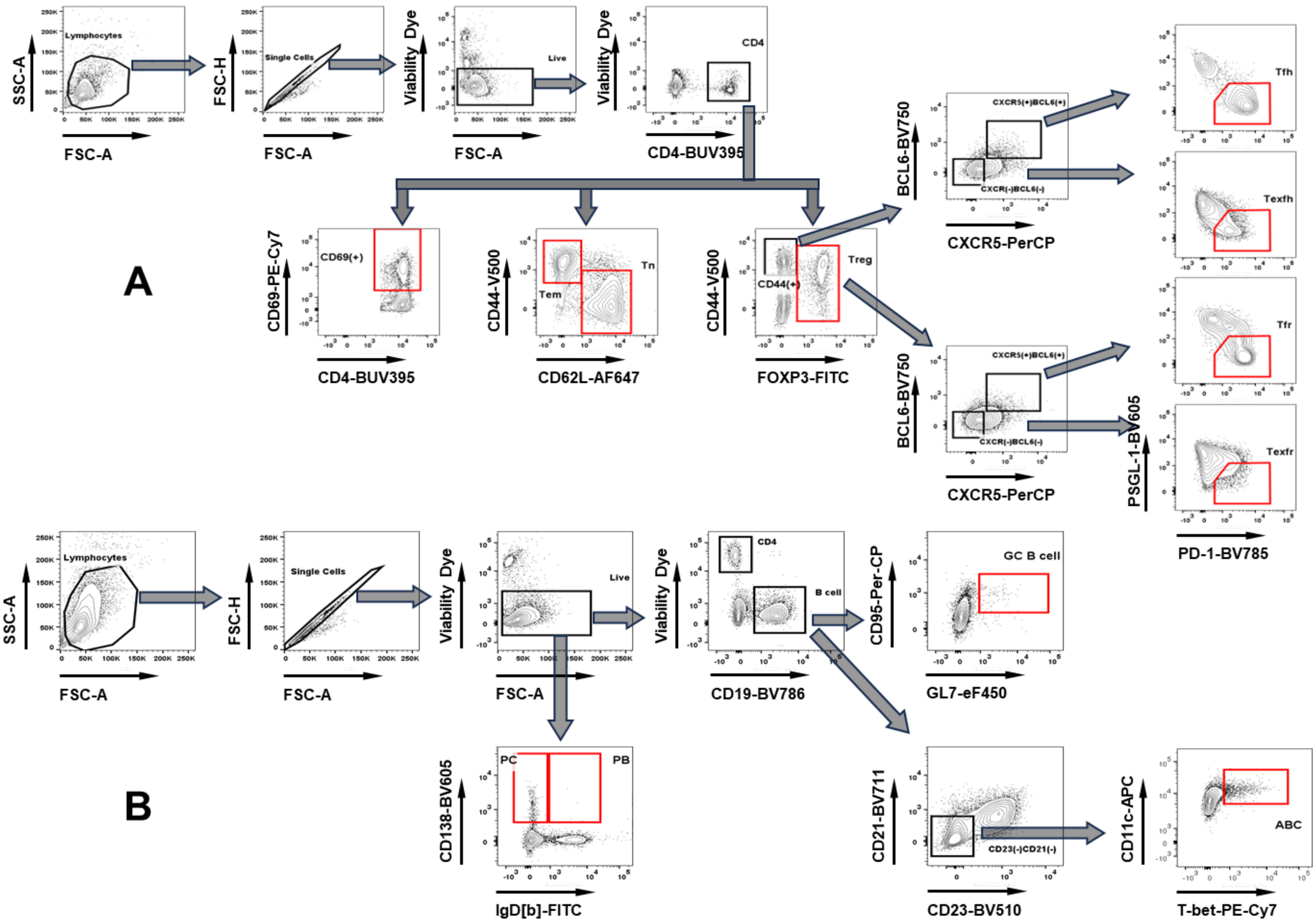

Supplemental Figure 2. Gating strategies for mouse CD4⁺ T cells (A) and B cells (B).

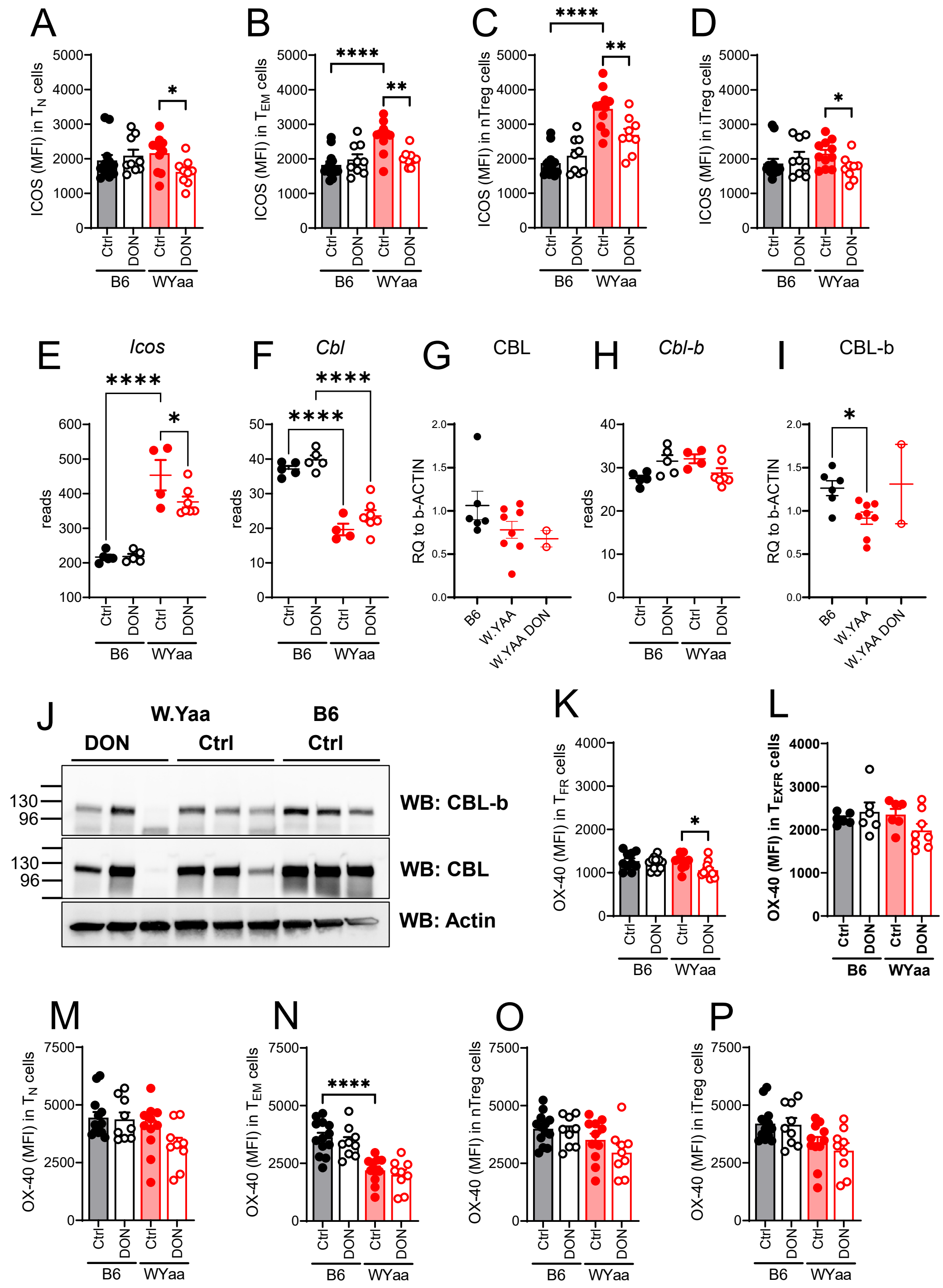

Supplemental Figure 3. DON reduced ICOS and OX-40 expression. ICOS expression on Tn (A), Tem (B), nTreg (C) and iTreg (D) cells. *Icos* (E), *Cbl* (F) and *Cbl-b* (H) expression (RNASeq dataset). CBL (G) and CBL-B (I) protein expression with representative Western blot image (J). OX-40 expression on Tfr (K), Texfr (L), Tn (M), Tem (N), nTreg (O) and iTreg (P) cells. Means ± SEM, N = 10–15 compared with Šídák's or Dunnet's (D) multiple comparisons tests. *: P < 0.05; **: P < 0.01; ***: P < 0.001; ****: P < 0.0001.

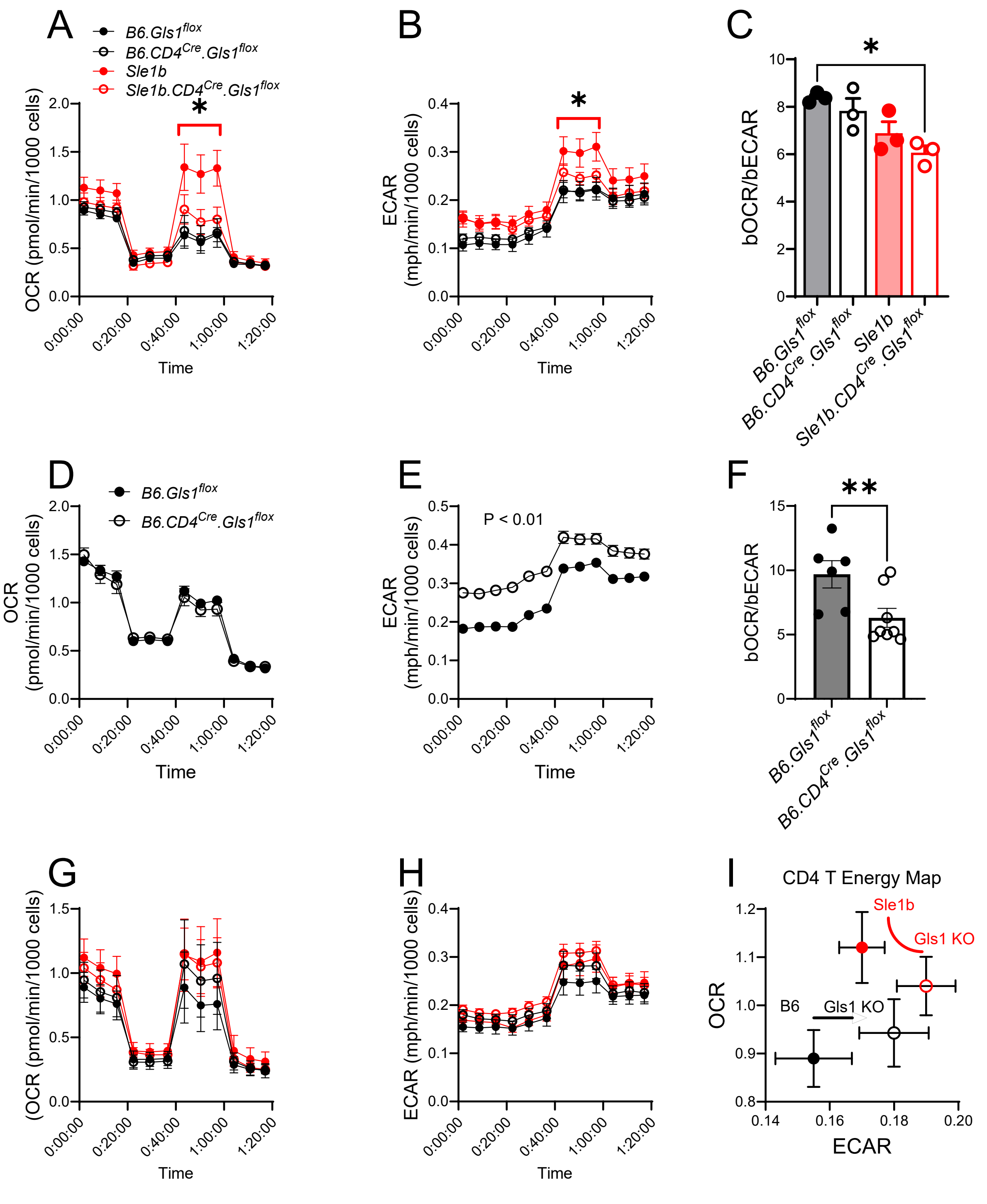

Supplemental Figure 4. *Gls1* CD4-KO increased glycolysis in CD4⁺ T cells. Mitochondrial stress assays were performed on CD4⁺ T cells from (A-C) untouched 9-12 months old mice (N = 3); (D-E) 5 weeks after cGVHD induction (N = 6-8); and (G-I) 7 weeks into a TD-recall response to NP-KLH immunization (N = 5). OCR (A, D, G) and ECAR (B, E, H). bOCR/bECAR ratio (C, F). I. Energy map. Mean + SEM. A and B: *t* tests for the 3 time points in the brackets between *Sle1b* and *Sle1b Gls1* CD4-Cre. E. t tests for each time point. C and F: Mann-Whitney tests.

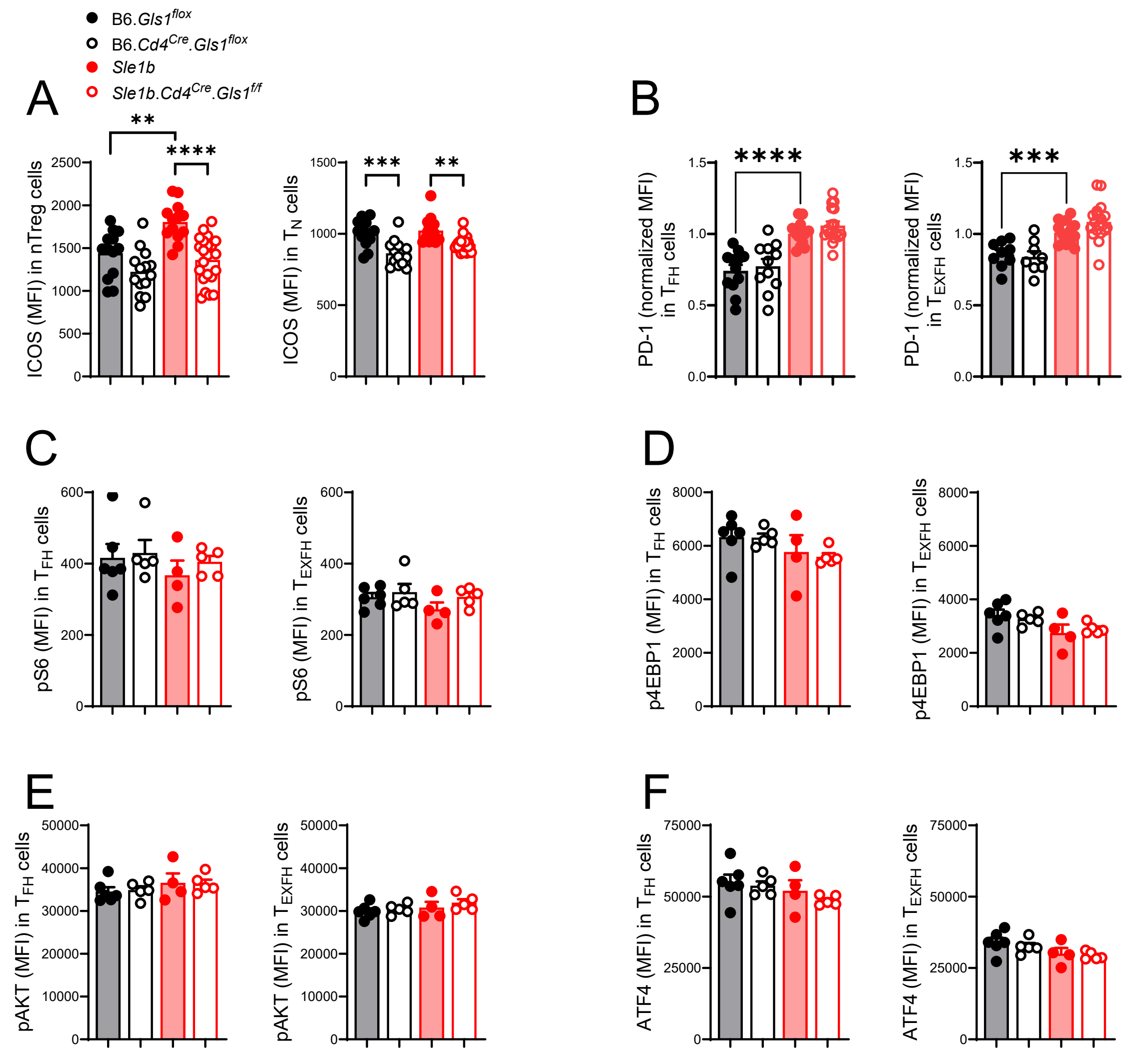

Supplemental Figure 5. CD4⁺ T cell phenotypes in aged *Gls1* CD4-KO mice. A. ICOS expression on nTreg (left) and Tn cells (right). B. PD-1 expression on Tfh and Texfh cells. N = 13-22 9–12 months old mice. Means + SEM, compared with Šídák's multiple comparisons tests. **: $P < 0.01$; ***: $P < 0.001$; ****: $P < 0.0001$. *Gls1* CD4-KO does not alter the expression of mTORC1 (B and C), mTORC2 (D) and GCN2 (E) in Tfh and Texfh cells. Means + SEM of MFI in 9–12 months old mice (N = 4-6).

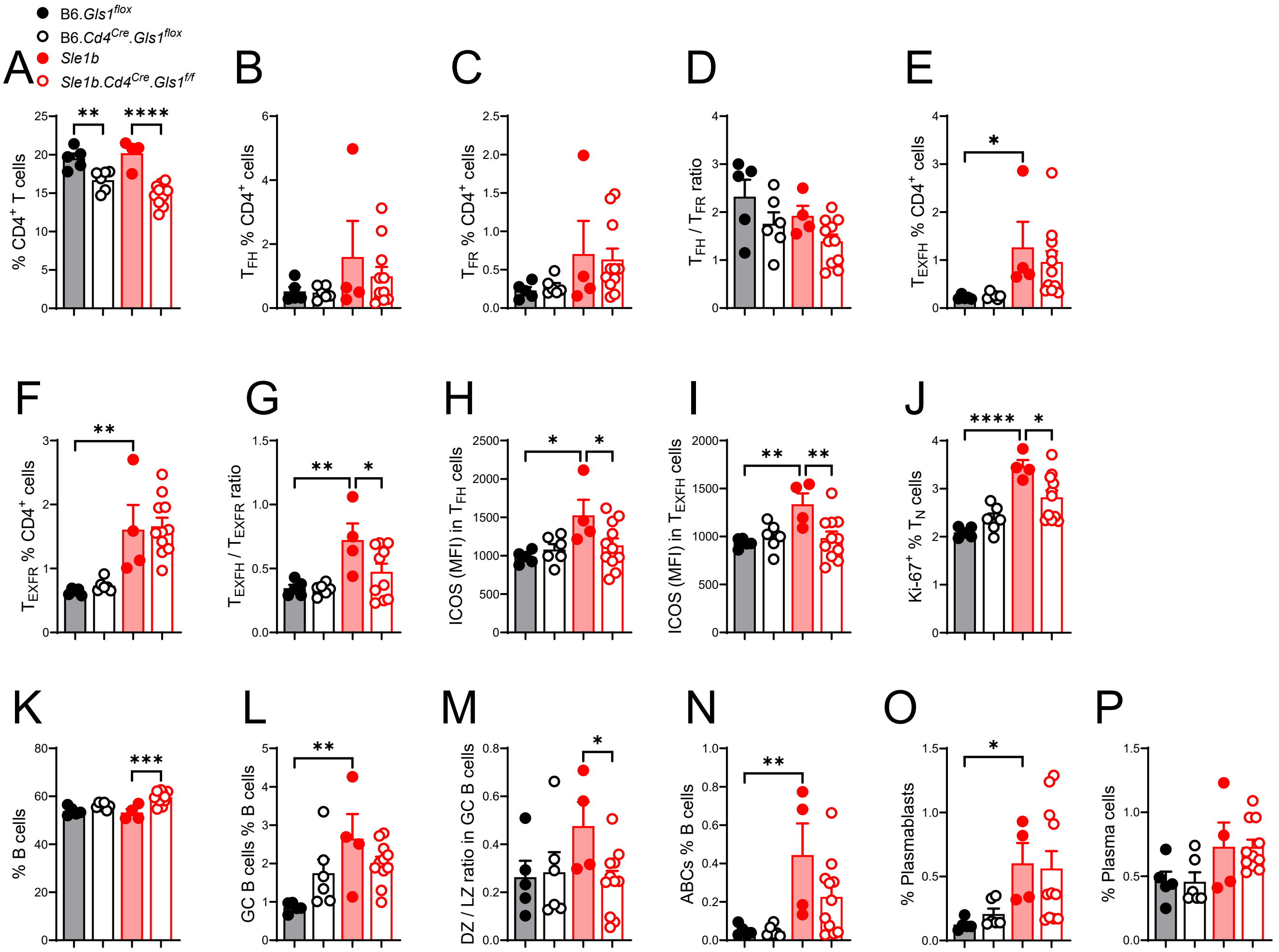

Supplemental Figure 6. Immunophenotypes of *Sle1b* mice were altered by *Gls1* CD4-KO before autoantibody production. Frequency of CD4⁺ T cells (A), Tfh (B), Tfr (C) cells and Tfh/Tfr ratio (D). Frequency of Texfh (E), Texfr (F) cells and Texfh/Texfr (G) ratio. ICOS expression on Tfh (H) and Texfh (I) cells. (J) Frequency of Ki-67⁺ Tn cells. Frequency of B cells (K) and GC B cells (L). (M) DZ/LZ ratio in GC B cells. Frequency of ABCs (N), plasmablasts (O) and plasma cells (P). N = 4–11 2-3 months old mice. Means ± SEM, compared with Šídák's multiple comparisons tests. *: $P < 0.05$; **: $P < 0.01$; ***: $P < 0.001$; ****: $P < 0.0001$.

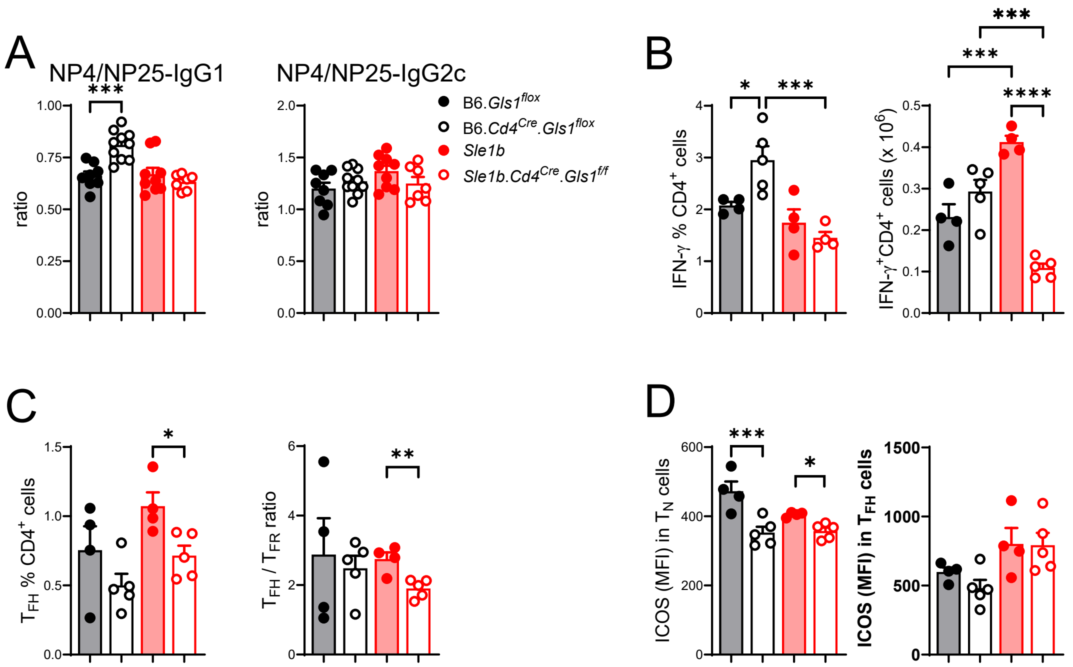

Supplemental Figure 7. *Gls1* CD4-KO altered the T-dependent humoral response. (A-B) Primary response to NP-KHL. (A) NP4/NP25 IgG1 and IgG2c ratios including values from week 1 of the recall response. (B) Frequency and number of IFN-γ$^+$ CD4$^+$ T cells. (C –D) Recall response 1 week after boost: (C) Frequency of Tfh cells and Tfh/Tfr ratio. (D) ICOS expression on Tn and Tfh cells. N = 4–10 2-3 months old mice. Means $\pm$ SEM, compared with Šídák's multiple comparisons tests. *: P < 0.05; **: P < 0.01; ***: P < 0.001; ****: P < 0.0001.

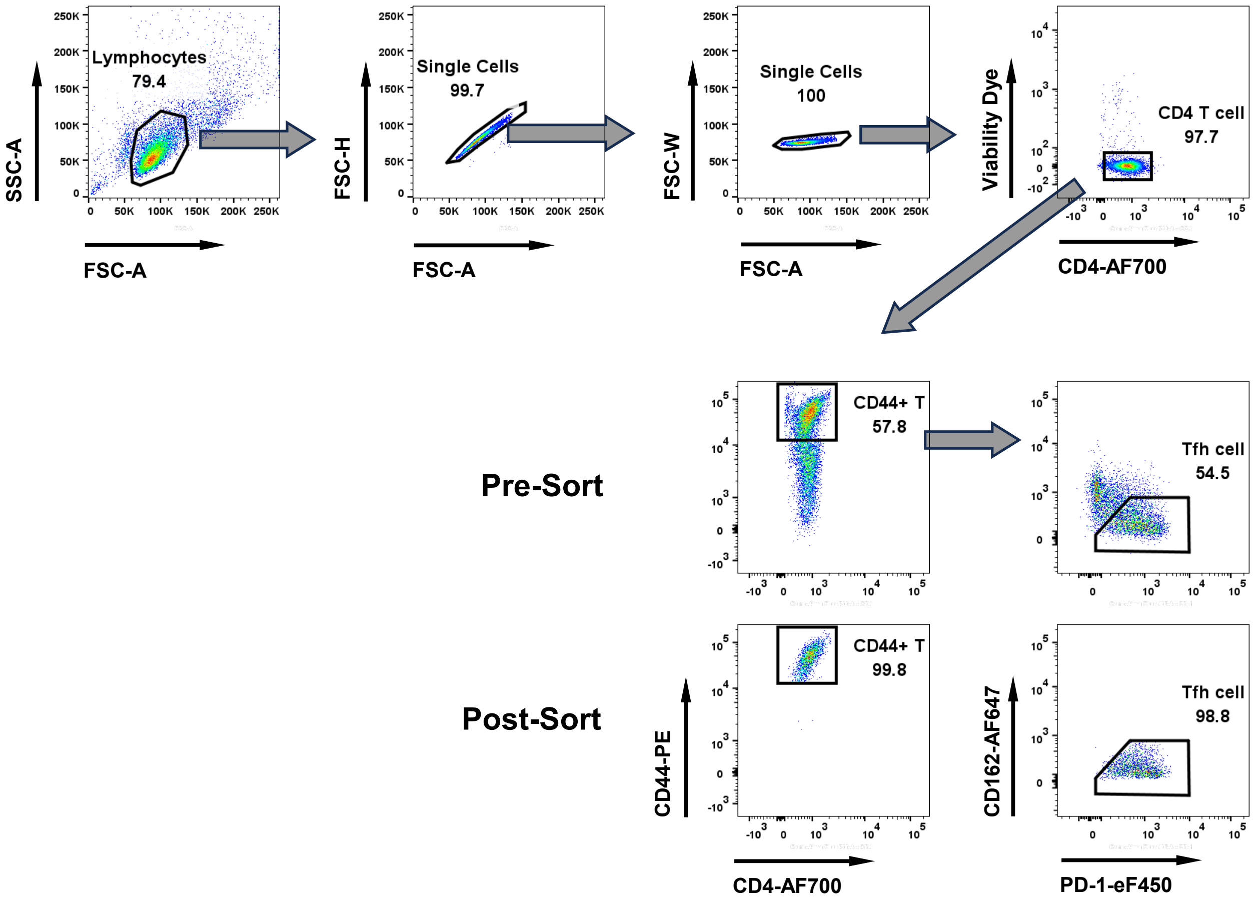

Supplemental Figure 8. Sorting strategy and efficiency for CD44$^+$ PSGL-1 (CD162)^{lo} PD-1$^+$ cells used as Tfh cells for RNASeq and metabolomics.
